# Supplementary material for: The association between power outages and cardiovascular and respiratory hospitalizations among US Medicare beneficiaries in 2018: A case-crossover study
Source: PLoS Med. 2026 Mar 12;23(3):e1004923. doi: 10.1371/journal.pmed.1004923 (PMC12994585; doi:10.1371/journal.pmed.1004923)
Supplement: S1 Table — (DOCX) [file pmed.1004923.s001.docx]

**Supplemental Table 1**: Distribution of power outage exposure by potential confounders for main analysis of county-level 8+ hour power outage exposure and CVD and respiratory hospitalizations in US 2018 Fee-For-Service Medicare beneficiaries.

| **Proportion of county-days with 8+ hour power outage affecting ≥1% of county customers by potential confounder quartiles** | | |
| --- | --- | --- |
| **Wind speed** | | |
|  | Quartile 1 | 0.009 |
|  | Quartile 2 | 0.009 |
|  | Quartile 3 | 0.010 |
|  | Quartile 4 | 0.022 |
| **Precipitation** | | |
|  | Quartile 1 | 0.013 |
|  | Quartile 2 | 0.014 |
|  | Quartile 3 | 0.012 |
|  | Quartile 4 | 0.011 |
| **Daily maximum temperature** | | |
|  | Quartile 1 | 0.022 |
|  | Quartile 2 | 0.012 |
|  | Quartile 3 | 0.009 |
|  | Quartile 4 | 0.008 |
