# Supplementary material for: The association between power outages and cardiovascular and respiratory hospitalizations among US Medicare beneficiaries in 2018: A case-crossover study
Source: PLoS Med. 2026 Mar 12;23(3):e1004923. doi: 10.1371/journal.pmed.1004923 (PMC12994585; doi:10.1371/journal.pmed.1004923)
Supplement: S2 Table — S2A Table: Rate ratios and 95% confidence intervals for the association between county-level 8+ hour power outage exposure and CVD and respiratory hospitalizations in US 2018 Fee-For-Service Medicare beneficiaries for outages affecting ≥1%, ≥3%, and ≥5% of county electrical customers. Estimates are from conditional Poisson regression models adjusted for daily wind speed, temperature, and precipitation. CVD, cardiovascular disease; Resp, respiratory disease. S2B Table: Rate ratios and 95% confidence intervals for effect modification analysis of the association between county-level 8+ hour power outage exposure and CVD and respiratory hospitalizations in US 2018 Fee-For-Service Medicare beneficiaries for outages affecting ≥1% of county electrical customers. Estimates are from conditional Poisson regression models adjusted for daily wind speed, temperature, and precipitation and stratified by effect modification categories. CVD, cardiovascular disease; Resp, respiratory disease. (DOCX) [file pmed.1004923.s002.docx]

**Supplemental Table 2A**: Rate ratios and 95% confidence intervals for the association between county-level 8+ hour power outage exposure and CVD and respiratory hospitalizations in US 2018 Fee-For-Service Medicare beneficiaries for outages affecting ≥1%, ≥3%, and ≥5% of county electrical customers. Estimates are from conditional Poisson regression models adjusted for daily wind speed, temperature, and precipitation.

CVD, cardiovascular disease; Resp, respiratory disease.

| Power outage cut point | Outcome type | Lag day 0 | Lag day 1 | Lag day 2 | Lag day 3 | Lag day 4 | Lag day 5 | Lag day 6 |
| --- | --- | --- | --- | --- | --- | --- | --- | --- |
| 1% | CVD | 1.002,  [0.99, 1.014] | 1.020,  [1.013, 1.026] | 1.023,  [1.014, 1.032] | 1.006,  [0.999, 1.012] | 0.988,  [0.98, 0.997] | 0.992,  [0.986, 0.998] | 1.009,  [0.997, 1.021] |
| 3% | CVD | 1.016,  [0.999, 1.032] | 1.027,  [1.018, 1.036] | 1.027,  [1.015, 1.039] | 1.009,  [1.001, 1.018] | 0.993,  [0.981, 1.005] | 0.995,  [0.987, 1.003] | 1.009,  [0.993, 1.025] |
| 5% | CVD | 1.025,  [1.005, 1.045] | 1.031,  [1.021, 1.042] | 1.029,  [1.014, 1.044] | 1.014,  [1.004, 1.024] | 1.000,  [0.986, 1.014] | 1.000,  [0.991, 1.01] | 1.010,  [0.991, 1.03] |
| 1% | Resp | 1.025,  [1.012, 1.038] | 1.017,  [1.01, 1.024] | 1.009,  [1.003, 1.016] | 1.004,  [0.995, 1.012] | 1.000,  [0.993, 1.007] | 0.998,  [0.992, 1.004] | 0.997,  [0.985, 1.01] |
| 3% | Resp | 1.052,  [1.034, 1.071] | 1.031,  [1.022, 1.04] | 1.013,  [1.004, 1.022] | 1.001,  [0.99, 1.012] | 0.997,  [0.988, 1.006] | 0.999,  [0.99, 1.007] | 1.004,  [0.987, 1.021] |
| 5% | Resp | 1.067,  [1.046, 1.089] | 1.039,  [1.029, 1.05] | 1.016,  [1.005, 1.027] | 1.001,  [0.988, 1.014] | 0.997,  [0.987, 1.008] | 1.001,  [0.991, 1.011] | 1.009,  [0.988, 1.029] |

**Supplemental Table 2B**: Rate ratios and 95% confidence intervals for effect modification analysis of the association between county-level 8+ hour power outage exposure and CVD and respiratory hospitalizations in US 2018 Fee-For-Service Medicare beneficiaries for outages affecting ≥1% of county electrical customers. Estimates are from conditional Poisson regression models adjusted for daily wind speed, temperature, and precipitation and stratified by effect modification categories.

CVD, cardiovascular disease; Resp, respiratory disease.

| Effect modification analysis | Category | Outcome type | Lag day 0 | Lag day 1 | Lag day 2 | Lag day 3 | Lag day 4 | Lag day 5 | Lag day 6 |
| --- | --- | --- | --- | --- | --- | --- | --- | --- | --- |
| Age | 75 and over | CVD | 1.008, [0.994, 1.023] | 1.019, [1.011, 1.027] | 1.019, [1.008, 1.03] | 1.003, [0.996, 1.01] | 0.989, [0.979, 1] | 0.993, [0.986, 1.001] | 1.009, [0.995, 1.024] |
| Age | Age 65 - 75 | CVD | 0.99, [0.97, 1.009] | 1.021, [1.011, 1.032] | 1.032, [1.017, 1.047] | 1.01, [1, 1.02] | 0.986, [0.972, 1] | 0.988, [0.978, 0.998] | 1.008, [0.989, 1.028] |
| Sex | Male | CVD | 1.007, [0.99, 1.025] | 1.018, [1.009, 1.027] | 1.017, [1.005, 1.03] | 1, [0.992, 1.009] | 0.986, [0.974, 0.998] | 0.991, [0.982, 1] | 1.008, [0.992, 1.025] |
| Sex | Female | CVD | 0.997, [0.982, 1.013] | 1.021, [1.013, 1.03] | 1.028, [1.016, 1.04] | 1.01, [1.002, 1.018] | 0.99, [0.979, 1.001] | 0.992, [0.984, 1.001] | 1.01, [0.994, 1.026] |
| Poverty | 1st quartile poverty | CVD | 0.985, [0.965, 1.004] | 1.02, [1.01, 1.031] | 1.03, [1.015, 1.045] | 1.001, [0.991, 1.011] | 0.973, [0.959, 0.988] | 0.988, [0.978, 0.998] | 1.031, [1.011, 1.051] |
| Poverty | 4th quartile poverty | CVD | 1.029, [0.996, 1.063] | 1.023, [1.006, 1.041] | 1.015, [0.991, 1.04] | 1.003, [0.986, 1.02] | 0.996, [0.973, 1.02] | 1.001, [0.984, 1.019] | 1.014, [0.983, 1.047] |
| DME | 1st quartile DME use | CVD | 0.997, [0.98, 1.014] | 1.027, [1.018, 1.035] | 1.033, [1.02, 1.045] | 1.002, [0.994, 1.011] | 0.975, [0.963, 0.987] | 0.986, [0.978, 0.995] | 1.023, [1.007, 1.041] |
| DME | 4th quartile DME use | CVD | 0.985, [0.946, 1.026] | 1.01, [0.988, 1.033] | 1.02, [0.99, 1.051] | 1.006, [0.984, 1.028] | 0.989, [0.961, 1.019] | 0.992, [0.97, 1.014] | 1.007, [0.967, 1.048] |
| Age | 75 and over | Resp | 1.032, [1.016, 1.048] | 1.02, [1.011, 1.028] | 1.009, [1.001, 1.017] | 1.001, [0.991, 1.011] | 0.996, [0.988, 1.004] | 0.994, [0.986, 1.001] | 0.992, [0.977, 1.008] |
| Age | Age 65 - 75 | Resp | 1.015, [0.995, 1.035] | 1.012, [1.001, 1.022] | 1.009, [0.999, 1.02] | 1.007, [0.994, 1.02] | 1.006, [0.996, 1.016] | 1.006, [0.996, 1.016] | 1.006, [0.986, 1.026] |
| Sex | Male | Resp | 1.026, [1.007, 1.045] | 1.018, [1.008, 1.027] | 1.01, [1, 1.02] | 1.003, [0.991, 1.015] | 0.997, [0.988, 1.006] | 0.991, [0.982, 1] | 0.986, [0.969, 1.004] |
| Sex | Female | Resp | 1.026, [1.009, 1.043] | 1.016, [1.007, 1.025] | 1.008, [1, 1.017] | 1.003, [0.993, 1.014] | 1.002, [0.994, 1.011] | 1.004, [0.996, 1.012] | 1.007, [0.991, 1.024] |
| Poverty | 1st quartile poverty | Resp | 1.023, [1.002, 1.046] | 1.012, [1.002, 1.023] | 1.003, [0.992, 1.014] | 0.997, [0.984, 1.011] | 0.995, [0.984, 1.006] | 0.996, [0.985, 1.006] | 0.998, [0.978, 1.019] |
| Poverty | 4th quartile poverty | Resp | 1.032, [0.997, 1.068] | 1.013, [0.994, 1.031] | 0.998, [0.979, 1.016] | 0.991, [0.969, 1.014] | 0.996, [0.978, 1.014] | 1.009, [0.991, 1.027] | 1.026, [0.992, 1.062] |
| DME | 1st quartile DME use | Resp | 1.03, [1.011, 1.049] | 1.019, [1.009, 1.028] | 1.008, [0.999, 1.018] | 1.001, [0.989, 1.013] | 0.997, [0.987, 1.006] | 0.995, [0.986, 1.004] | 0.994, [0.976, 1.012] |
| DME | 4th quartile DME use | Resp | 0.986, [0.945, 1.028] | 0.989, [0.966, 1.012] | 0.991, [0.969, 1.014] | 0.994, [0.968, 1.021] | 0.997, [0.975, 1.02] | 1, [0.978, 1.023] | 1.003, [0.963, 1.046] |
