# Supplementary material for: The association between power outages and cardiovascular and respiratory hospitalizations among US Medicare beneficiaries in 2018: A case-crossover study
Source: PLoS Med. 2026 Mar 12;23(3):e1004923. doi: 10.1371/journal.pmed.1004923 (PMC12994585; doi:10.1371/journal.pmed.1004923)
Supplement: S3 Table — Estimates are from conditional Poisson regression models adjusted for daily wind speed, temperature, and precipitation. (DOCX) [file pmed.1004923.s003.docx]

**Supplemental Table 3**: Rate ratios and 95% confidence intervals for the association between county-level 8+ hour power outage exposure and cardiovascular- and respiratory-related hospitalizations in US 2018 Medicare Fee-For-Service beneficiaries on anomalously hot and anomalously cold days. Estimates are from conditional Poisson regression models adjusted for daily wind speed, temperature, and precipitation.

|  | **CVD hot** | **CVD cold** | **Respiratory hot** | **Respiratory cold** |
| --- | --- | --- | --- | --- |
| 8+ hour power outage | RR = 1.03  95% CI: [1.02, 1.04] | RR = 1.04  95% CI: [1.03 1.05] | RR = 1.00  95% CI: [1.00, 1.01] | RR = 1.04  95% CI: [1.02, 1.06] |
| Anomalous temperature day | RR = 1.00  95% CI: [0.99, 1.00] | RR = 1.05  95% CI: [1.04, 1.06] | RR = 1.03  95% CI: [1.01, 1.05] | RR = 1.07  95% CI: [1.06, 1.08] |
| Power outage and anomalous temperature day interaction | RR = 1.06  95% CI:[1.02, 1.10] | RR = 0.96  95% CI: [0.91, 1.01] | RR = 1.02  95% CI: [0.97, 1.07] | RR = 0.91  95% CI: [0.85, 0.97] |
