# Supplementary material for: The association between power outages and cardiovascular and respiratory hospitalizations among US Medicare beneficiaries in 2018: A case-crossover study
Source: PLoS Med. 2026 Mar 12;23(3):e1004923. doi: 10.1371/journal.pmed.1004923 (PMC12994585; doi:10.1371/journal.pmed.1004923)
Supplement: S1 Fig — Estimates are from conditional Poisson regression models adjusted for daily wind speed, temperature, and precipitation. (DOCX) [file pmed.1004923.s004.docx]

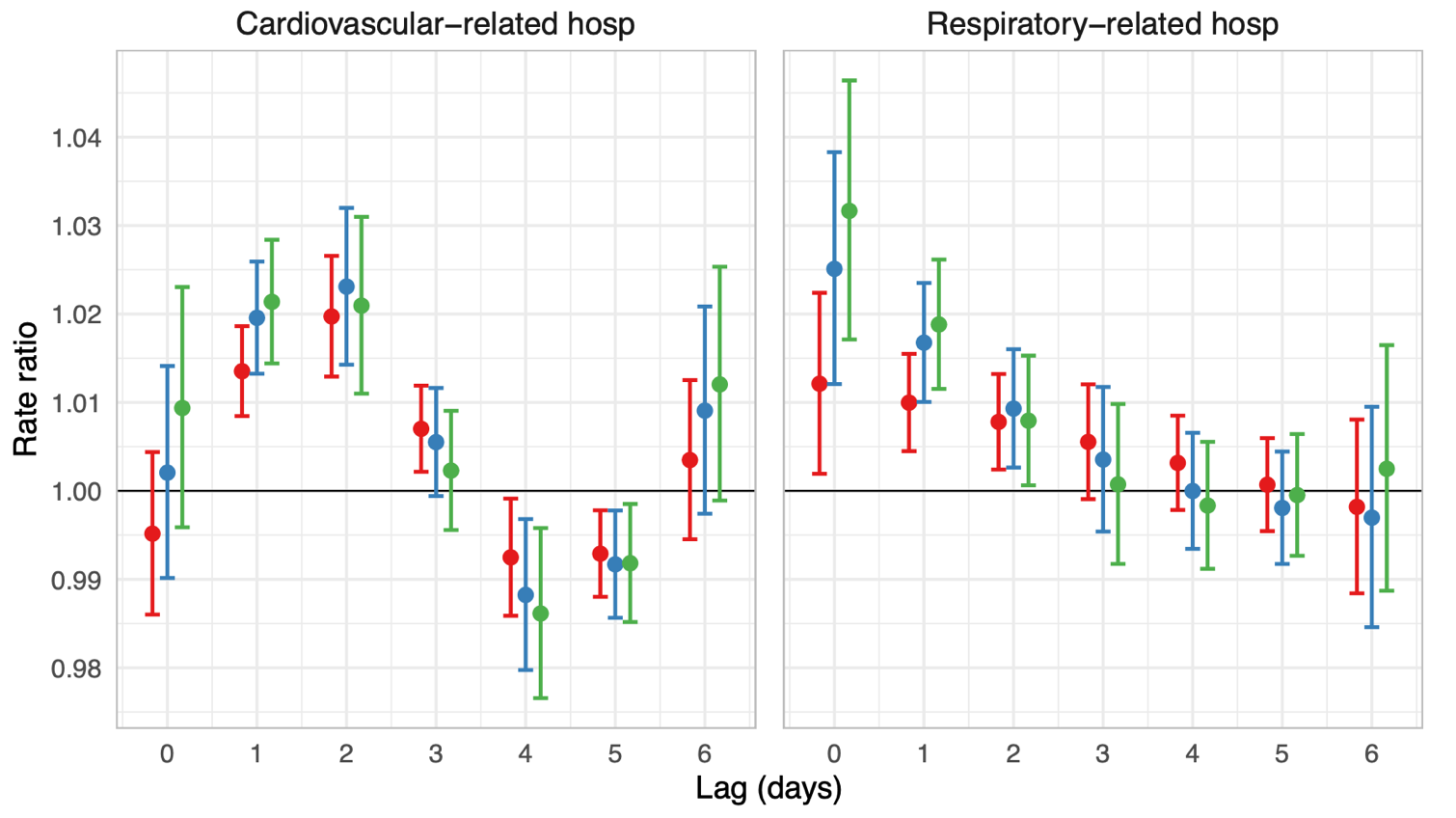
**
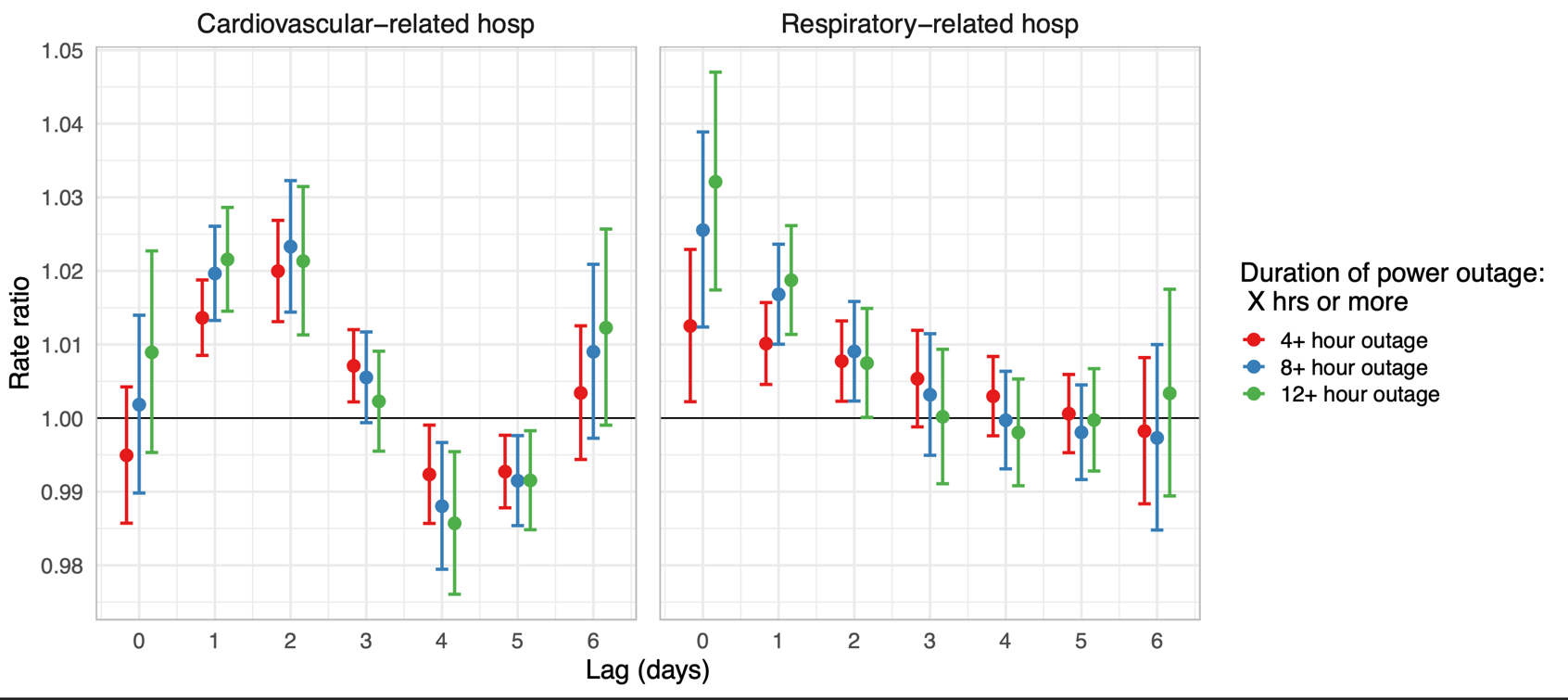
**

**Supplemental Figure 1**: Rate ratios and 95% confidence intervals (bars) for the association between county-level 8+ hour power outage exposure and CVD and respiratory hospitalizations in US 2018 Fee-For-Service Medicare beneficiaries for 4+, 8+, and 12+ hour power outages affecting ≥1% of county customers. Estimates are from conditional Poisson regression models adjusted for daily wind speed, temperature, and precipitation.
