# Supplementary material for: The association between power outages and cardiovascular and respiratory hospitalizations among US Medicare beneficiaries in 2018: A case-crossover study
Source: PLoS Med. 2026 Mar 12;23(3):e1004923. doi: 10.1371/journal.pmed.1004923 (PMC12994585; doi:10.1371/journal.pmed.1004923)

**Supplemental Figure 2**: Rate ratios and 95% confidence intervals (shading) for the association between county-level daily number of hours without power and cardiovascular- and respiratory-related hospitalizations in US 2018 Medicare Fee-For-Service beneficiaries. Estimates are from conditional Poisson regression models adjusted for daily wind speed, temperature, and precipitation. Results are from the best fitting model tested based on qAIC comparison, with a linear relationship between number of hours without power and respiratory hospitalizations or CVD hospitalizations, and 4 degrees of freedom on the lag dimension.


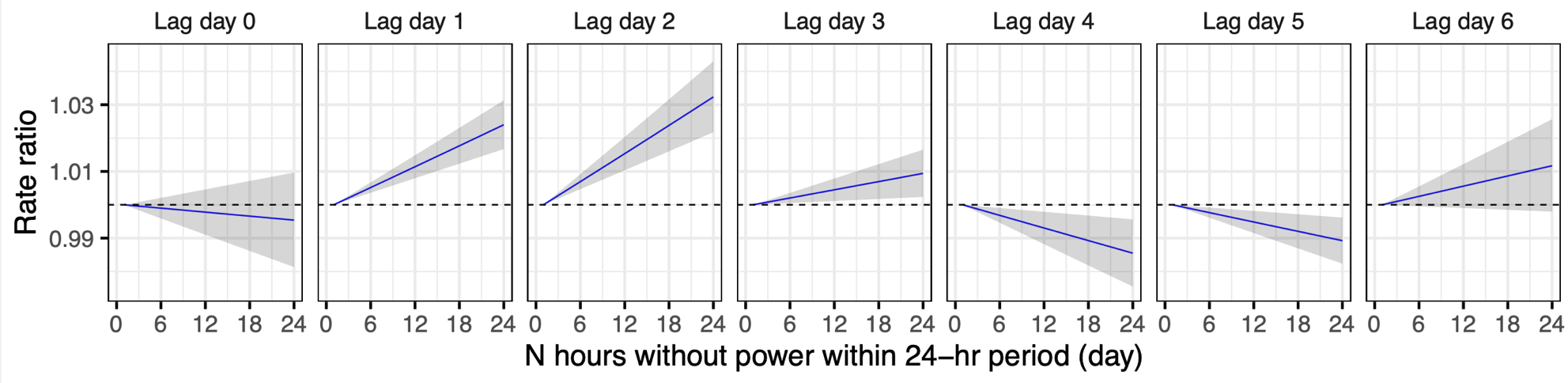
Cardiovascular-related hospitalizations

Respiratory-related hospitalizations


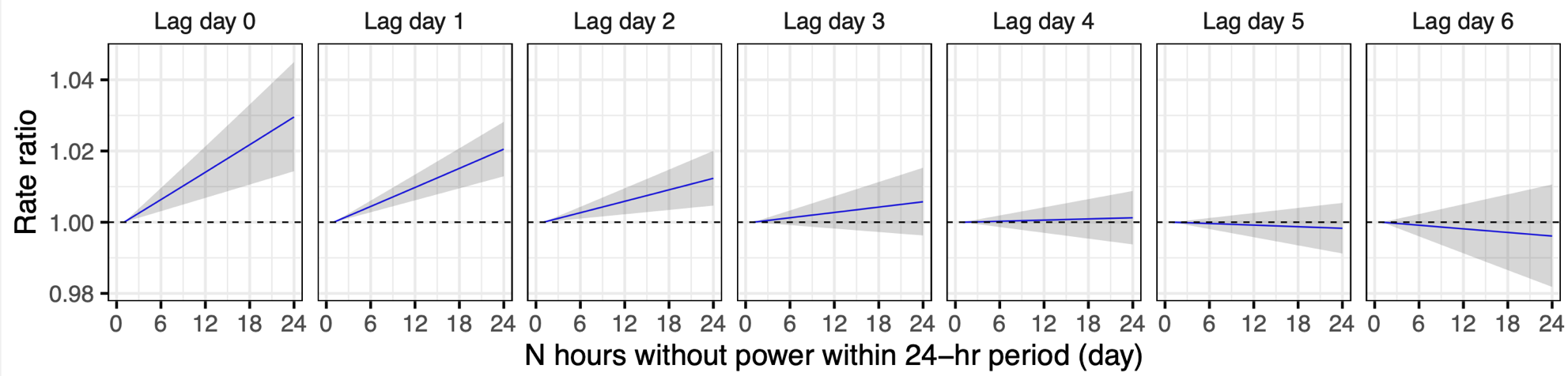

Supplement: S2 Fig — Estimates are from conditional Poisson regression models adjusted for daily wind speed, temperature, and precipitation. Results are from the best fitting model tested based on qAIC comparison, with a linear relationship between number of hours without power and respiratory hospitalizations or CVD hospitalizations, and 4 degrees of freedom on the lag dimension. (DOCX) [file pmed.1004923.s005.docx]
