# Supplementary material for: The association between power outages and cardiovascular and respiratory hospitalizations among US Medicare beneficiaries in 2018: A case-crossover study
Source: PLoS Med. 2026 Mar 12;23(3):e1004923. doi: 10.1371/journal.pmed.1004923 (PMC12994585; doi:10.1371/journal.pmed.1004923)
Supplement: S3 Fig — In this sensitivity analysis, instead of last observation carried forward, some missing values were replaced with 0. Estimates are from conditional Poisson regression models adjusted for daily wind speed, temperature, and precipitation. (DOCX) [file pmed.1004923.s006.docx]

**
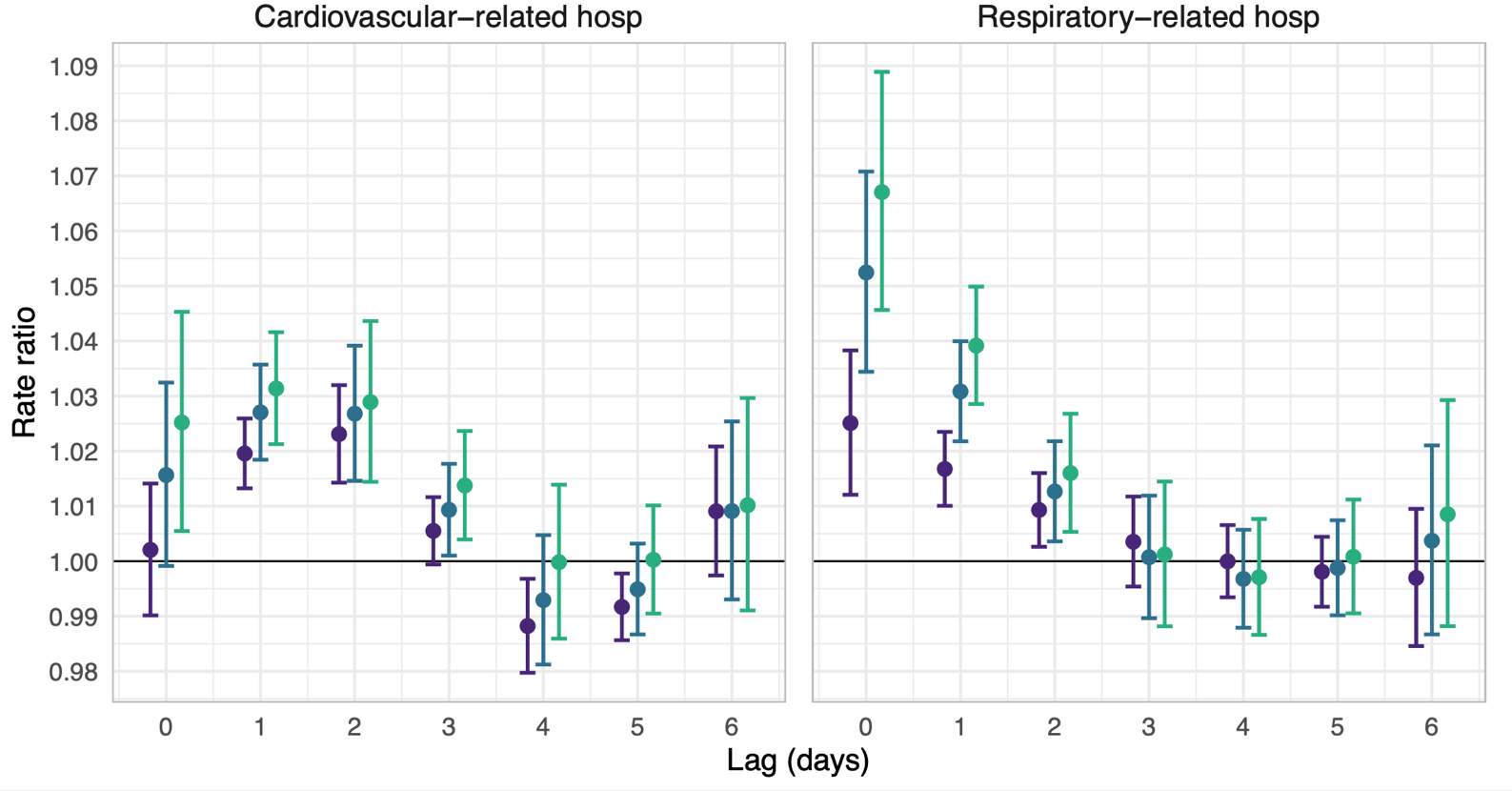
**

**
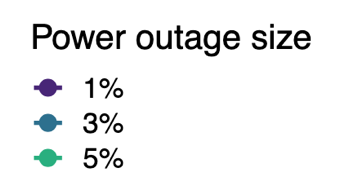
**

≥ 1%

≥ 3%

≥ 5%

**Supplementary Figure 3**: Rate ratios and 95% confidence intervals (bars) for the association between county-level 8+ hour power outage exposure and cardiovascular- and respiratory-related hospitalizations in US 2018 Medicare Fee-For-Service beneficiaries for outages affecting ≥1%, ≥3%, and ≥5% of county electrical customers. In this sensitivity analysis, instead of last observation carried forward, some missing values were replaced with 0. Estimates are from conditional Poisson regression models adjusted for daily wind speed, temperature, and precipitation.
