# Supplementary material for: The association between power outages and cardiovascular and respiratory hospitalizations among US Medicare beneficiaries in 2018: A case-crossover study
Source: PLoS Med. 2026 Mar 12;23(3):e1004923. doi: 10.1371/journal.pmed.1004923 (PMC12994585; doi:10.1371/journal.pmed.1004923)
Supplement: S4 Fig — Estimates are from conditional Poisson regression models adjusted for daily wind speed, temperature, and precipitation. (DOCX) [file pmed.1004923.s007.docx]

**
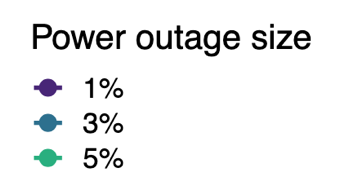
**
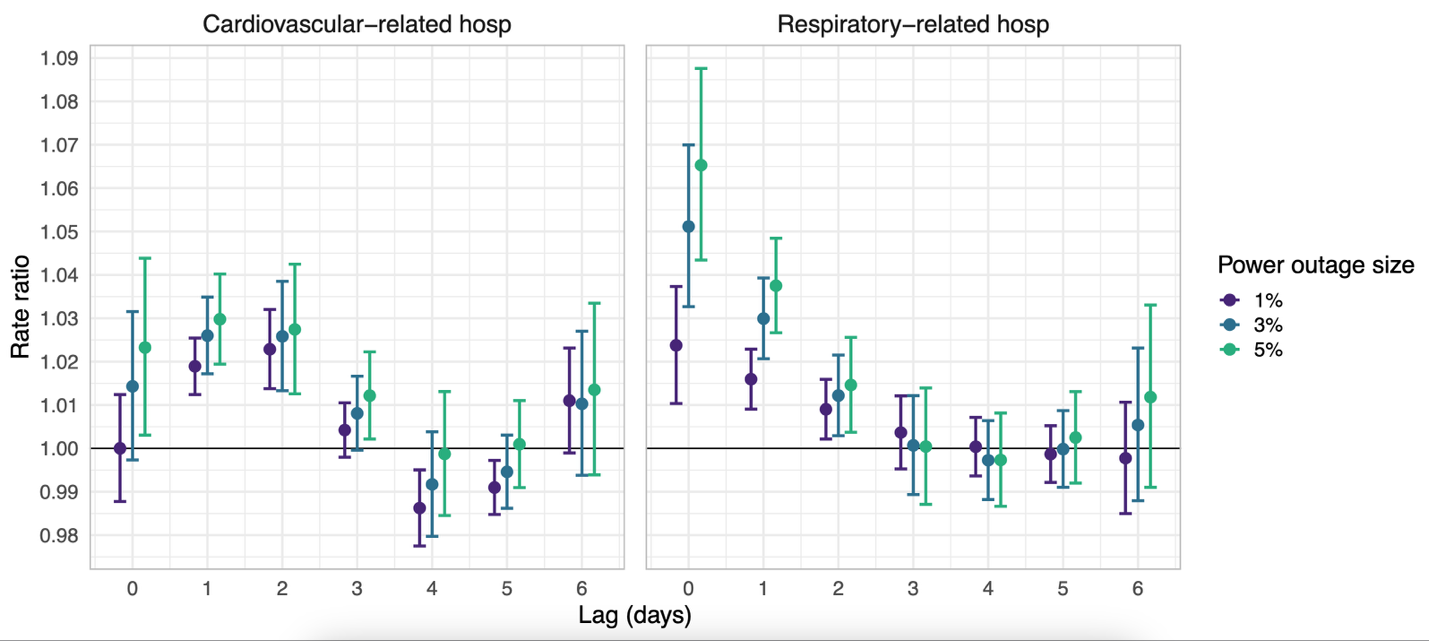

**Supplemental Figure 4**: Rate ratios and 95% confidence intervals (bars) for the association between county-level 8+ hour power outage exposure and cardiovascular- and respiratory-related hospitalizations in US 2018 Medicare Fee-For-Service beneficiaries for outages affecting ≥1%, ≥3%, and ≥5% of county electrical customers, in counties with 20% of data missing or less. Estimates are from conditional Poisson regression models adjusted for daily wind speed, temperature, and precipitation.

≥ 1%

≥ 3%

≥ 5%
