# Supplementary material for: The association between power outages and cardiovascular and respiratory hospitalizations among US Medicare beneficiaries in 2018: A case-crossover study
Source: PLoS Med. 2026 Mar 12;23(3):e1004923. doi: 10.1371/journal.pmed.1004923 (PMC12994585; doi:10.1371/journal.pmed.1004923)
Supplement: S5 Fig — (DOCX) [file pmed.1004923.s008.docx]

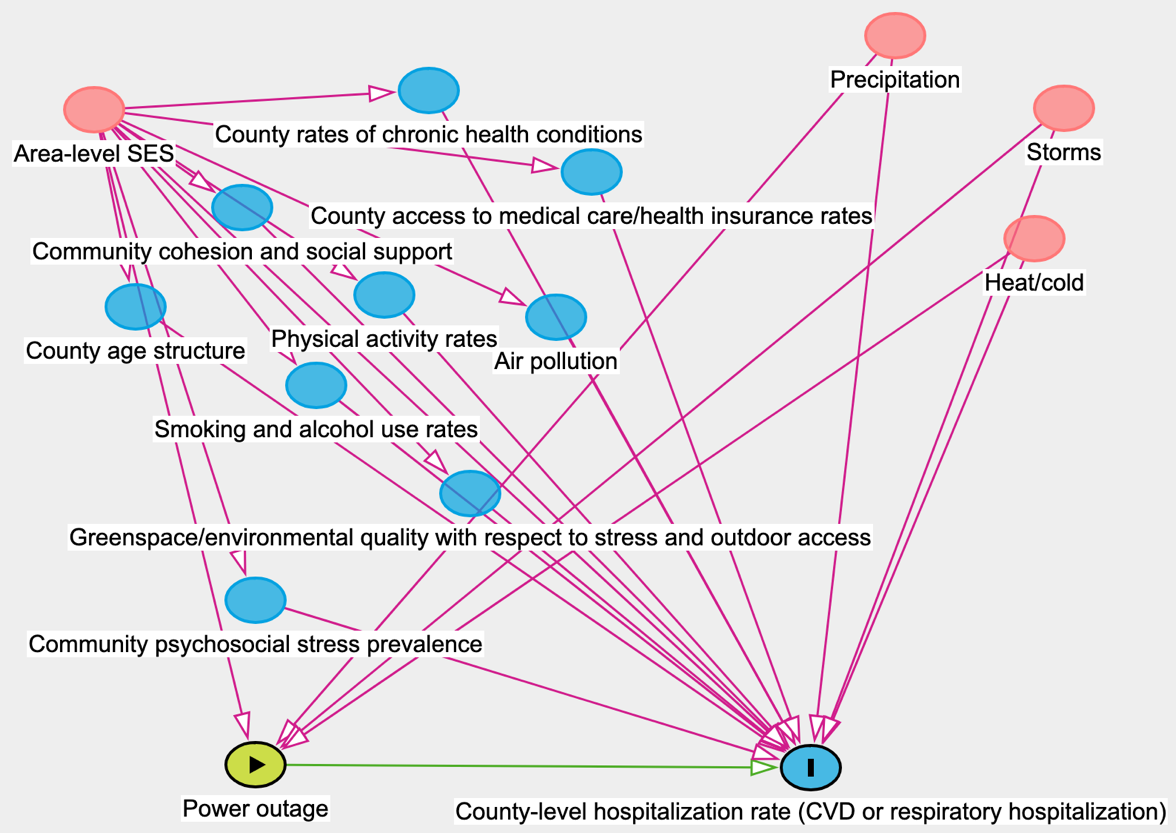


**Supplemental Figure 5**: Directed acyclic graph describing hypothesized causal relationships between power outage, hospitalizations among older adults, and potential confounders.
