# Supplementary material for: The association between power outages and cardiovascular and respiratory hospitalizations among US Medicare beneficiaries in 2018: A case-crossover study
Source: PLoS Med. 2026 Mar 12;23(3):e1004923. doi: 10.1371/journal.pmed.1004923 (PMC12994585; doi:10.1371/journal.pmed.1004923)
Supplement: S6 Fig — Estimates are from conditional Poisson regression models adjusted for daily wind speed, temperature, precipitation and wildfire PM2.5. (DOCX) [file pmed.1004923.s009.docx]

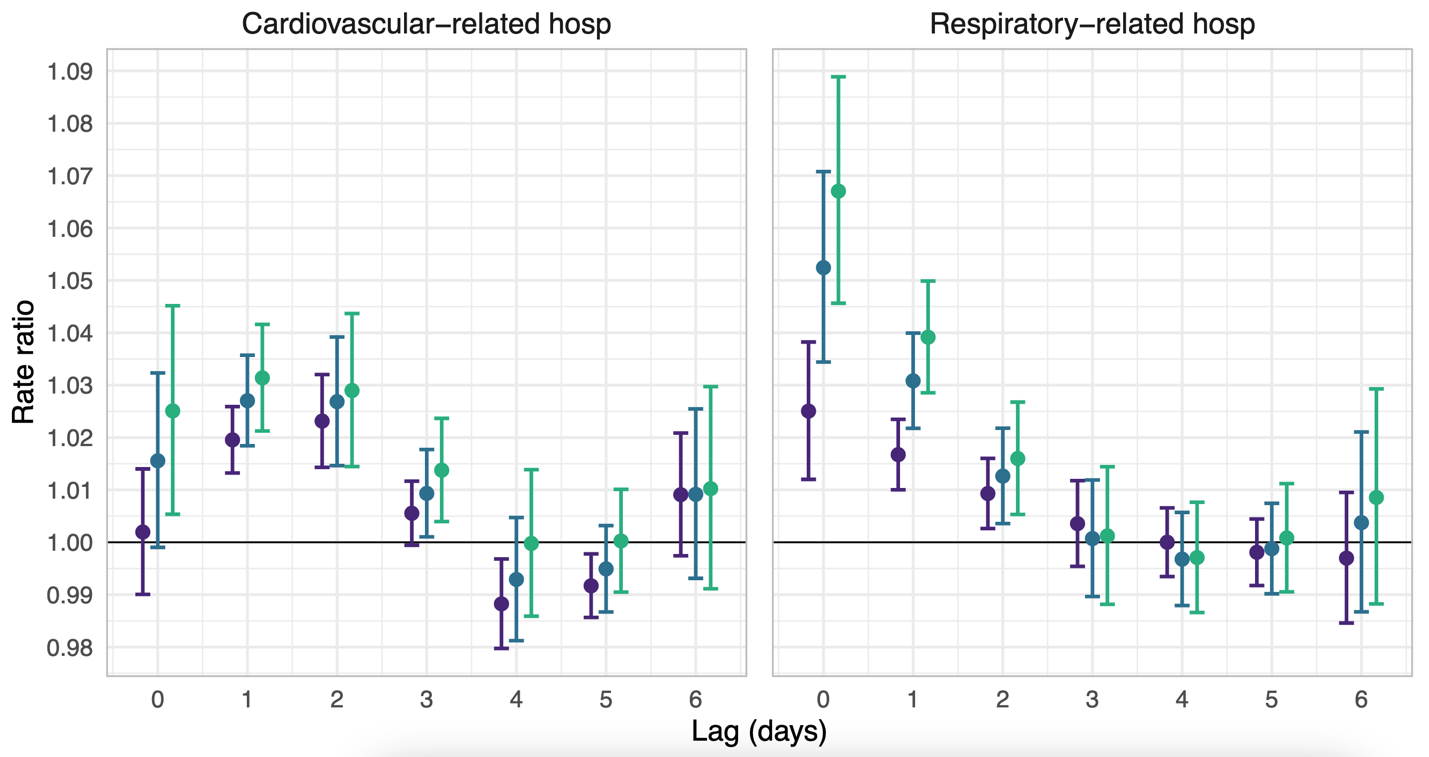


**
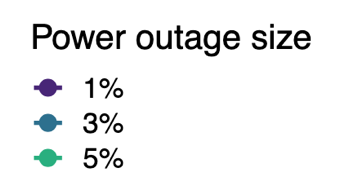
**

≥ 1%

≥ 3%

≥ 5%

**Supplemental Figure 6**: Rate ratios and 95% confidence intervals (bars) for the association between county-level 8+ hour power outage exposure and cardiovascular- and respiratory-related hospitalizations in US 2018 Medicare Fee-For-Service beneficiaries for outages affecting ≥1%, ≥3%, and ≥5% of county electrical customers. Estimates are from conditional Poisson regression models adjusted for daily wind speed, temperature, precipitation, and wildfire PM_2.5_.
