# Supplementary material for: The association between power outages and cardiovascular and respiratory hospitalizations among US Medicare beneficiaries in 2018: A case-crossover study
Source: PLoS Med. 2026 Mar 12;23(3):e1004923. doi: 10.1371/journal.pmed.1004923 (PMC12994585; doi:10.1371/journal.pmed.1004923)
Supplement: Attachment — Submitted filename: mcbrien.pdf [file pmed.1004923.s011.pdf]

This paper describes the association between power outages and hospitalisations in the United States.

I was asked for a methodological report and I interpret that to include all aspects of the design and conduct of the study.

Points of detail

Page 6 Using last observation carried forward seems rather arbitrary. My initial thought was that I would have tried to average some readings before and after the missing data period. If the missingness is related to power outages which occurred during the period of missingness imputing what happened before will be biased.

Page 7 I assume the person had to be living in the same county on the control day as the power outage day. Can this be clarified?

Page 7 Could people enter the models twice if their county experienced more than one event? If they could how was this included in the models?

Pages 7 and 8 The authors have fitted slightly different models to cardiovascular disease (CVD) and to respiratory disease. I wonder whether this is optimal if there is going to be a comparison between the conditions.

Page 8 I am afraid I have very little knowledge about Medicare or Medicaid. Are these universal, that is, does everyone over age 65 become included?

Page 9 A mean of 7 with a standard deviation of 29 is crying out for expression also as quantiles given the obvious skew. I would suggests 5% and 95% as well as the usual quartiles.

Page 10 Looking at Figure 2 and the confidence intervals there I find it difficult to agree about the effect at 6 days for CVD. Unless there is a strong theoretical argument for supposing that the effect returns after six days I would focus on the earlier period where the confidence intervals do not include the null unlike 6 days.

Page 10 Is the statement comparing the outage sizes based on a formal test? I do not see this as too important since the pattern of results over the early days and the dose–response effect are quite compelling.

Page 11 A really minor point but I would call out Figure 2 immediately after the first respiratory results (as done for CVD).

Supplementary Figure 2 This seems to be to reinforce my comments about 6 days for CVD. Incidentally there is a typo cardiovascular not cardiovasular.

Points of more substance

Generalisability

I had to read page 9 several times to convince myself that I had understood just how many power outages there are in the United States. The map was helpful here. For context I cannot remember of the several decades of my adult life ever experiencing an outage of 8+ hours and I do not think that is a memory problem. My country does experience some long power outages but usually in only in rural areas in very severe winter weather. In an international context what is the authors' message to our governments? Is it 'This could happen to you?'.

DME use

There appears to be no mention in the text of effect modification for CVD To be fair Figure 3 does present a display. At the very least it should be discussed in the main text. This seems to me an opportunity for using CVD as a negative control.

Summary

Mostly points for clarification about the analysis. I have no issues with the core strategy.

Michael Dewey
