# Supplementary material for: The association between power outages and cardiovascular and respiratory hospitalizations among US Medicare beneficiaries in 2018: A case-crossover study
Source: PLoS Med. 2026 Mar 12;23(3):e1004923. doi: 10.1371/journal.pmed.1004923 (PMC12994585; doi:10.1371/journal.pmed.1004923)
Supplement: Attachment — Submitted filename: response_to_reviewers_sep_2_a.docx [file pmed.1004923.s012.docx]

We would like to thank the Editor and Reviewers for the thoughtful comments and suggestions. We have addressed these reviews, greatly improving our manuscript. Below please find our responses to the specific comments.

Comments from the reviewers:

Reviewer #1: This is a study on a novel research question. Generally the study appears to be well conducted and reported. Particularly, Figure 2 is very clear and illustrated the results beautifully. Thank you! My methodological comments are:

Thank you for your comments, Reviewer 1. We very much appreciate your feedback and contributions.

1. It appears the study used ecological case-cross over design, where the 'ecological' part is that the unit of analysis is based on county rather than individuals. If my interpretation is correct please be explicit about the study's ecological nature, and discuss the limitations arises from this, notably ecological fallacy and potentially individual-level time varying confounding.

This is a good point, thank you. We added the following sentences to the first paragraph of the study limitations section (page 20, study limitations, paragraph 1):

“In this study, we assessed county-level power outage exposure since no national finer-resolution power outage exposure data are available. We also aggregated ZCTA-level counts of hospitalizations to the county level. Our ecological study was subject to the ecological fallacy and potential time-varying confounding bias, meaning that the relationship between power outage and hospitalizations observed at the county level may not hold at the individual level, despite its theoretical plausibility and community concerns about power outage and adverse health outcomes (50–52). At this time, no individual-level nationwide power outage data are available.”

2. More details are required on the selection of 'control' days. It was reported as 'matching on county, day of week, and month'. In that case the control days would be at least 1 year apart from the 'case' days. How far apart are the case and control days are? Would it not be an issue that with year difference, demographic shift would play a non-negligible role?

Thank you for this comment. Multiple reviewers requested that we clarify how we selected control days. We would like to clarify that our study only spanned one year (2018), and therefore, case days were also automatically matched to control days on year as well, in addition to the rest of the matching factors. We have updated this paragraph and included some citations to justify the principles we used in selecting control days (page 7, study design paragraph 1, starting at line 5):

“For each case day (i.e., a county-day with a non-zero hospitalization count), we selected control days from the same county in the month and on the same day of the week. Since our study included only days from 2018, control days were also matched on the same year. This matching controlled for time-invariant confounders such as county-level socioeconomic factors that might influence both power outage and cardiorespiratory hospitalization rates, as well as long-term, seasonal, and day-of-week trends. We tested for overdispersion in the outcome by running a quasi-Poisson model, but detected none, and thus proceeded with a traditional Poisson model.”

3. Consider splitting some text in Statistical Analysis into a separate section as 'Study design' for clarity.

Thank you for this suggestion. We have added a new section called ‘Study design’ (page 7) which includes the following text, and we have labelled the next section ‘Statistical analysis’.

“We used a time-stratified case-crossover design with a conditional Poisson model (30) to analyze the association between daily county-level power outage exposure and CVD or respiratory hospitalization rates. We modeled CVD and respiratory hospitalizations separately because heat, cold, loss of power to medical devices, and dehydration may affect these outcomes differently (31–33). For each case day (i.e., a county-day with a non-zero hospitalization count), we selected control days from the same county in the month and on the same day of the week. Since our study included only days from 2018, control days were also matched on the same year. This matching controlled for time-invariant confounders such as county-level socioeconomic factors that might influence both power outage and cardiorespiratory hospitalization rates, as well as long-term, seasonal, and day-of-week trends. We tested for overdispersion in the outcome by running a quasi-Poisson model, but detected none, and thus proceeded with a traditional Poisson model.”

4. Consider adding a negative control outcome (e.g. infectious disease?) - this could add confidence to the findings given the ecological nature.

Thank you for this suggestion. We considered this when conceptualizing the paper. However, we have been unable to find a good negative control outcome for such an analysis that would add confidence in the results. For negative control outcomes to effectively add confidence, the confounders that influence the primary outcome of interest and the negative control outcome must be the same and modelled correctly in both the primary analysis and negative control analysis. Our present analysis could be confounded by severe weather and temperature, and so a negative control analysis could theoretically help us detect residual confounding from these variables. Unfortunately, we don’t believe that the relationships between temperature and infectious disease and temperature and CVD and respiratory hospitalizations are analogous enough that a negative control analysis would add confidence to our results. For example, after elevated temperature, it would likely take longer for infectious disease rates to change compared to often-observed same-day impacts on cardiorespiratory hospitalizations. Infectious disease hospitalizations might be reduced due to severe weather in the same way that CVD and respiratory hospitalizations are, but seasonality is likely a stronger driver for infectious disease compared to cardiorespiratory hospitalizations. Additionally, to correctly model the relationship between power outage and infectious disease, our modelling strategy would need to account for disease transmission. Thus, while we are open to conducting a negative control analysis if we could find an appropriate negative control outcome, we have opted not to proceed with infectious disease as an endpoint.

Reviewer #2: This paper describes the association between power outages and hospitalisations in the United States. I was asked for a methodological report and I interpret that to include all aspects of the design and conduct of the study.

Points of detail

Page 6 Using last observation carried forward seems rather arbitrary. My initial thought was that I would have tried to average some readings before and after the missing data period. If the missingness is related to power outages which occurred during the period of missingness imputing what happened before will be biased.

To produce the power outage exposure data, we started with counts of customers out by utility, where multiple utilities could exist in a county. We then aggregated these counts to the county level. When there were fewer than 4 hours of information missing at the sub-county level, we used last observation carried forward (LOCF). However, this accounted for < 0.3% of total observations. There was also substantial missing data spanning longer than 4 hours of time. We did not LOCF in that case of longer-duration missingness. In a recent manuscript, we described our strategies to handle missing data in the power outage exposure dataset and assessed the impact of missingness on epidemiologic results like the one under consideration here:

<https://journals.lww.com/environepidem/fulltext/2025/08000/assessing_bias_in_measuring_power_outage_exposure.4.aspx>

We agree that LOCF was somewhat arbitrary, but it was easily implemented, used a portion of existing data to replace missing values, and affected < 0.3% of the data. In response to the reviewer comment regarding LOCF, we have run a sensitivity analysis using a different strategy, Instead of using LOCF, we replaced data missing for fewer than 4 hours with 0 (no exposure, the mean, median, and mode value of exposure in the dataset).

Our results from this sensitivity analysis were consistent with our main results:

**Supplemental Figure 3**: Rate ratios and 95% confidence intervals for the association between county-level 8+ hour power outage exposure and cardiovascular- and respiratory-related hospitalizations in US 2018 Medicare Fee-For-Service beneficiaries for outages affecting ≥1%, ≥3%, and ≥5% of county electrical customers. In this sensitivity analysis, instead of last observation carried forward, some missing values were replaced with 0. Estimates are from conditional Poisson regression models adjusted for daily wind speed, temperature, and precipitation.

≥ 1%

≥ 3%

≥ 5%

Page 7 I assume the person had to be living in the same county on the control day as the power outage day. Can this be clarified?

The first reviewer also found our text about our selection of control days unclear. We assumed that people were living in the same county on the control day as on the power outage day. Because our control days were selected within the same month as the case days, we considered this a fair assumption, and we think any movement would not have impacted effect estimates. We clarified this with the following text: (page 7, study design paragraph 1, starting at line 5):

“For each case day (i.e., a county-day with a non-zero hospitalization count), we selected control days from the same county in the month and on the same day of the week. Since our study included only days from 2018, control days were also matched on the same year. This matching controlled for time-invariant confounders such as county-level socioeconomic factors that might influence both power outage and cardiorespiratory hospitalization rates, as well as long-term, seasonal, and day-of-week trends. We tested for overdispersion in the outcome by running a quasi-Poisson model, but detected none, and thus proceeded with a traditional Poisson model.”

Page 7 Could people enter the models twice if their county experienced more than one event? If they could how was this included in the models?

Our study was a case-crossover analysis, and we aggregated counts to the county level to use a conditional Poisson model for the analysis. This is statistically equivalent to a case-crossover analysis with a logistic regression model. Because it was a case-crossover, people could be in the models twice if they were hospitalized twice or more during the study period, and they might have also been included in the models if they were hospitalized on a selected control day. Their presence in the model was dependent on if they experienced the outcome rather than the exposure.

Pages 7 and 8 The authors have fitted slightly different models to cardiovascular disease (CVD) and to respiratory disease. I wonder whether this is optimal if there is going to be a comparison between the conditions.

We have indeed fit two different models for each outcome. Our goal in doing so was to correctly model the relationship between exposure, outcome, and confounders to best estimate the effect of power outage on CVD and respiratory outcomes. We hypothesized that power outage would cause cardiovascular and respiratory hospitalizations because of heat or cold exposure, loss of electricity to medical equipment, mobility devices and elevators, and refrigeration, water, and communication devices. There are different relationships, for example, between heat and cold, dehydration, and CVD vs. respiratory conditions. This could result in the lagged effect of heat exposure on CVD being different than the lagged effect of heat on respiratory conditions. Furthermore, studies of other environmental exposures (e.g., air pollution, temperature) indicate that the highest risk period for CVD is different than that for respiratory outcomes (i.e., lags 0-2 vs. one week, respectively). We, therefore, chose to model these outcomes differently and separately because of these differing hypothesized relationships.

The third reviewer also asked us to address these differences in the discussion, and so we have included the following text clarifying this (page 17, paragraph 2, line 1):

“We hypothesized that power outages may lead to CVD and respiratory hospitalizations in older adults due to increased heat exposure, cold exposure, stress, and loss of electricity to life-sustaining medical devices and mobility aids. Power outages may also cause changes in indoor air quality when dehumidifiers, air purifiers, and ventilation systems lose power. The lagged effects of CVD and respiratory hospitalizations due to power outage likely differ, since the lagged effects of high or low temperature and air pollution differ for CVD versus respiratory disease (35, 41, 42).”

Page 8 I am afraid I have very little knowledge about Medicare or Medicaid. Are these universal, that is, does everyone over age 65 become included?

Thank you for this comment. We clarify this both here and in the manuscript. Most people living in the US above 65 are eligible for Medicare coverage and are enrolled (97%, https://www.ssa.gov/history/pdf/WhatMedicareMeant.pdf ). Very few people are not eligible – for example, non-residents are not eligible. However, those eligible have the option to choose from two different kinds of Medicare coverage: Fee-For-Service or Advantage. Our dataset only includes Fee-For-Service Medicare enrollees, which consists of about half of the US population over 65 or approximately 23 million adults (65+). More work is needed to determine how Fee-For-Service enrollees differ from Medicare Advantage enrollees demographically (ongoing work in our group) but a few non-peer-reviewed publications have described these differences (see the citations we include below). Advantage enrollees may be more vulnerable to health effects from power outage compared to Fee-For-Service enrollees based on demographic differences reported in these white papers, such as income or educational differences, measures of social risk, rates of chronic health conditions, and healthcare utilization among Advantage enrollees. In summary, the people included in our manuscript may be less vulnerable to health effects from power outage compared to the other half of the older adult US population, who are not as well represented here. We’ve added a paragraph to the methods section about this population and how it compares to the overall population, as well as text to the discussion about how this may impact the generalizability of our results (page 4, Methods, Study population):

“Our study population included Medicare Fee-For-Service beneficiaries aged 65+ and enrolled for at least one month between January 1, 2018, and December 31, 2018. From the Medicare Beneficiary Summary File (MBSF), we obtained age, sex, Medicaid-eligibility status, and county of residence for all beneficiaries. Medicare Fee-For-Service beneficiaries comprise approximately 50% of the US population aged 65+. This population may have fewer chronic health conditions, more education and income, and less healthcare utilization compared to Medicare Advantage beneficiaries (25, 26).”

Discussion (page 20, final paragraph):

“Next, power outages may be more common in high social vulnerability communities (5). High social vulnerability counties may have been over-represented in the exposed group in our study, and the health effects of power outages may be stronger in these places. This means that our effect estimates may not be generalizable to counties with lower social vulnerability, and we may have overestimated the effect of power outage on hospitalization. At the same time, our study population was comprised of Medicare Fee-For-Service beneficiaries, who may be less vulnerable to health effects from power outages due to higher individual-level SES and fewer chronic health conditions compared to Medicare Advantage beneficiaries, which may have led to underestimation of the population-level effect.”

Page 9 A mean of 7 with a standard deviation of 29 is crying out for expression also as quantiles given the obvious skew. I would suggests 5% and 95% as well as the usual quartiles.

Thank you, this point was brought up by another reviewer as well. We now report the IQR and 5^th^ and 95^th^ quantiles (page 11, paragraph 2):

“We included 2,161 US counties, covering 71.1% of older adult Medicare Fee-For-Service beneficiaries aged 65+ (N = 23,645,101). These counties experienced a median of 2 (IQR = 4; 5^th^ percentile = 0; 95^th^ percentile = 15) 8+ hour power outages affecting ≥1% of customers in 2018 (**Figure 1**)”.

Page 10 Looking at Figure 2 and the confidence intervals there I find it difficult to agree about the effect at 6 days for CVD. Unless there is a strong theoretical argument for supposing that the effect returns after six days I would focus on the earlier period where the confidence intervals do not include the null unlike 6 days.

Thank you for bringing this up. There is some evidence in the literature of week-later delayed effects of heat on CVD hospitalizations, but we were already on the fence about how to interpret that last lag, even before this reviewer comment. We have removed our former interpretation. The manuscript now reads (page 12, CVD hospitalizations, Main analysis):

“In our main analysis, we used a case-crossover design with a conditional Poisson model to test the association between 8+ hour power outage and emergency CVD hospitalization rates up to 1 week after power outage exposure. We found increases in CVD-related hospitalizations 1-3 days after power outage exposure (**Figure 2**).”

Page 10 Is the statement comparing the outage sizes based on a formal test? I do not see this as too important since the pattern of results over the early days and the dose–response effect are quite compelling.

Thank you for bringing this up. We think it’s a very good point. No, these comparisons are not based on a test. They are only based on the results from our models and our interpretation of them. It is true that the confidence intervals for the smaller vs. larger outages do overlap, so it may not be appropriate to say that the effect is higher with the larger outages. Instead, we now comment only on the difference in point estimates and mention the degree of overlap in the CIs (page 12, final paragraph):

“We also analyzed larger outages affecting ≥3% or ≥5% of county customers. Outages affecting ≥3% of county customers had a stronger association with next-day CVD hospitalization rates than outages affecting ≥1% of county customers (Figure 2). Outages affecting ≥5% of customers were associated with even higher next-day hospitalization rates compared to outages affecting ≥3% or ≥1% of county customers, though the confidence intervals for all of these point estimates overlapped. For outages affecting ≥3% of county customers, the day after outage CVD rates were 2.7% (95% CI: 1.8%, 3.6%) higher than on days after no exposure. For outages affecting ≥5% of the population, rates were 3.1% (95% CI: 2.1%, 4.2%) higher than on days after no exposure.”

Page 11 A really minor point but I would call out Figure 2 immediately after the first respiratory results (as done for CVD).

Thank you – we added this. The text now reads (page 13, Respiratory hospitalizations, Main analysis):

“In our main analysis testing the effect of 8+ hour power outage exposure on emergency respiratory hospitalization rates, we found same-day increases in respiratory-related hospitalizations, as well as increases on lag days 1 and 2 (**Figure 2**).”

Supplementary Figure 2 This seems to be to reinforce my comments about 6 days for CVD. Incidentally there is a typo cardiovascular not cardiovasular.

Thank you – we fixed this typo and changed the 6-day lag interpretation as is described in the previous comment.

Points of more substance

Generalisability

I had to read page 9 several times to convince myself that I had understood just how many power outages there are in the United States. The map was helpful here. For context I cannot remember of the several decades of my adult life ever experiencing an outage of 8+ hours and I do not think that is a memory problem. My country does experience some long power outages but usually in only in rural areas in very severe winter weather. In an international context what is the authors’ message to our governments? Is it ‘This could happen to you?’.

Thank you for bringing our attention to this paragraph, which we have updated. Counties were considered exposed to power outage if ≥1% of the population was without power for 8 consecutive hours or more. This means that only a very small proportion of the population needed to experience an 8+ hour outage for the county to be categorized as exposed. Given the wide differences in power outage exposure globally, we have opted to simply more accurately describe our exposure variable and allow readers to interpret the relevance of our findings in their particular context. The text now reads (page 11, results, first paragraph):

“We included 2,161 US counties, covering 71.1% of older adult Medicare Fee-For-Service beneficiaries aged 65+ (N = 23,645,101). These counties experienced a median of two (IQR = 4; 5^th^ percentile = 0; 95^th^ percentile = 15) 8+ hour power outages affecting ≥1% of customers in 2018 (**Figure 1**). Because counties were considered exposed to an 8+ hour power outage if ≥1% of customers were without power for 8+ hours, it was not the case that individuals experienced a median of two 8+ hour outages; rather, a small portion of customers in these counties were likely affected by 8+ hour outages”.

We also added the following sentence to the limitations (page 21, paragraph 2):

“This was also US-based study. Electrical grid infrastructure, social cohesion, and healthcare access vary significantly by country, and it is unclear whether our results generalize outside the United States. Other studies are needed to evaluate the health impacts of power outages internationally.”

DME use

There appears to be no mention in the text of effect modification for CVD To be fair Figure 3 does present a display. At the very least it should be discussed in the main text. This seems to me an opportunity for using CVD as a negative control.

To directly address CVD in addition to respiratory hospitalizations, we have added the following (page 15, effect modification, final paragraph):

“The effect of power outage on CVD hospitalizations was not modified by county-level DME use.”

The suggestion to use CVD (one of our main two outcomes) as a negative control is not clear to us. Could the reviewer clarify? For a discussion of use of negative control outcomes in our analysis, please see our response in comment 4.

Summary

Mostly points for clarification about the analysis. I have no issues with the core strategy.

Michael Dewey

Thank you for your clear and helpful review Dr. Dewey! We sincerely appreciate your feedback.

Reviewer #3: This study investigates the association between power outages and emergency hospitalizations for cardiovascular and respiratory diseases among older adults in the US. The authors assembled the first nationwide dataset of power outage exposure and linked it with Medicare claims data from 23 million beneficiaries aged 65 and older at county level. Using a case-crossover design with a conditional Poisson model, they analyzed the lagged effects (up to 1 week) of outages on hospitalization rates. Overall, the study addresses a critical gap in understanding the health impacts of power outages in the US, which are becoming more frequent due to climate change. However, I have several concerns regarding exposure assessment, confounding, and result interpretation.

(1) This study does not have exposure data at a fine spatial resolution such as census tract or ZIP code level. Power outage exposure is assessed at the county level, meaning many individuals within a county may not have actually experienced a power outage. The definition of exposure (≥1% of customers without power for 8+ hours) may have led to substantial exposure misclassification.

Thank you for this comment. Yes, this is true, and we share your concern. Unfortunately, the best nationwide power outage data available for the US are at the county level. In order to determine when a county was exposed to power outage, we needed to pick some threshold after which to consider a county exposed. Because of the inherent exposure misclassification involved in our exposure assessment strategy, we also conducted secondary analyses for which we used two other definitions of power outage exposure, considering counties exposed if ≥3 and ≥5% of customers were without power for 8+ hours. We found larger effect sizes when we increased the cut point, an expected de-attenuation for less non-differential exposure misclassification.

We have revised the text in the statistical analysis section to highlight the reason for our secondary analyses of these larger power outages and address the exposure misclassification concern (page 6, paragraph 2):

“Therefore, in our primary analysis, we considered a county-day exposed to power outage if ≥1% of county customers were without power for 8+ consecutive hours, a definition that may have substantial exposure misclassification (i.e., up to 99% of customers may be unexposed). To address this, we conducted a secondary analysis for which we assessed power outage exposure based on higher cut points of customers without power (≥3%, ≥5%), scenarios with less exposure misclassification.”

We’ve also included text on this issue in the study limitations (page 20, paragraph 2):

“In our main analysis, we considered a county-day exposed to power outage if ≥1% of county customers were without power for 8+ consecutive hours, a definition that may have substantial exposure misclassification (i.e., up to 99% of customers may be unexposed). This misclassification likely biased study results towards the null, but the magnitude of bias remains unknown. When we assessed power outage exposure based on higher cut points of customers without power (≥3%, ≥5%), scenarios with less exposure misclassification, the observed effect estimates were stronger. Future studies could collaborate with utilities to obtain finer-resolution power outage data or use satellite imagery to identify exact outage boundaries of long-duration outages lasting into the night (55, 56) to improve exposure assessment.”

(2) The [PowerOutage.us](http://PowerOutage.us) (POUS) dataset is incomplete, with many counties missing up to 50% of data. The authors used a simple method to impute county-days with missing 4 or fewer hours, but it is unclear how the remaining missing values were handled. Further discussion is needed on how missing data may have biased the results.

Thank you for this point. We were also concerned about the effects of missing data on our results before we began this project, and so we conducted simulations to test the effect of missing data on the results of this paper. That paper describing our simulations results was not published at the time we submitted the present manuscript. It has since been peer-reviewed and published; here is a link, which discussing in detail our strategy for handling missing data and the impacts that this strategy has on the results of an epidemiologic study, including this one:

<https://journals.lww.com/environepidem/fulltext/2025/08000/assessing_bias_in_measuring_power_outage_exposure.4.aspx>

In our methods section, we referenced this study and explained how we handled missing data and why (page 6, first paragraph):

“Substantial exposure data were missing from the POUS dataset. The POUS dataset was created using web scraping, and some utilities did not have websites, or their websites were offline during part or all of the study period. Previously, we conducted a simulation study where we treated missing data as no power outage exposure, because no exposure was the median value in the dataset (28). When doing so in simulations, missing data biased the results of a study like ours towards the null. When a small percentage (approximately <15%) of hours within each county (county-hours) were missing exposure data, the bias was minimal (37). When larger amounts (approximately >50%) were missing, the bias was substantial. To balance generalizability and bias in the present study, we excluded counties with >50% of county-hours missing in the POUS data (n=907 counties). On average, the remaining counties were missing data for 7% of county-hours. When we included counties were missing 4 or fewer hours of consecutive exposure data, we carried forward the last observation to impute those hours. Our final analytic dataset included counties in 48 states (all counties in HI and AK were excluded) and 2,161 counties, covering 71.1% of 2018 Fee-For-Service Medicare beneficiaries (N = 23,622,770).”

We also reference our simulation study in the study limitations section (page 21, paragraph 3):

“Finally, the POUS dataset we used to assess exposure is missing substantial data. We excluded counties missing more than 50% of customer-hours in 2018 to balance generalizability and bias from missing data based on our prior simulation study (28)”.

Finally, we have now added an additional sensitivity analysis only including counties with less than 20% missing data, in response to the reviewer’s comment below (statistical analysis, page 9, final paragraph):

“Because of the large proportion of missing data in the POUS dataset, we conducted an additional sensitivity analysis limiting our analysis to counties with 20% of data missing or less”

And added the following to the results (sensitivity analyses, page 11):

≥ 1%

≥ 3%

≥ 5%

“Restricting to counties with 80% or higher coverage (compared to 50% or higher in our main analyses) did not change our results (**Supplemental Figure 4**)”

**Supplemental Figure 4**: Rate ratios and 95% confidence intervals for the association between county-level 8+ hour power outage exposure and cardiovascular- and respiratory-related hospitalizations in US 2018 Medicare Fee-For-Service beneficiaries for outages affecting ≥1%, ≥3%, and ≥5% of county electrical customers, in counties with 20% of data missing or less. Estimates are from conditional Poisson regression models adjusted for daily wind speed, temperature, and precipitation.

(3) The study uses different sources for the number of affected customers and the total number of customers. This inconsistency may introduce bias in estimating the proportion of the population affected by power outages. The authors should justify this approach or explore alternative sources that ensure consistency.

Because we identified the customer counts from POUS as inaccurate and inconsistent, we opted to use alternative data sources to estimate customer counts by county. We have added additional justification about the unreliability of POUS customer counts (page 5, exposure assessment, paragraph 1):

“Utilities rarely provide total counts of customers served, and thus, these data are often unreliable from POUS. POUS captures counts of customers served when available but inconsistencies and gaps mean estimates provided by POUS often differ by orders of magnitude across months and years. Instead of relying on these unstable estimates, we 2013-2018 American Community Survey estimates of the number of households and establishments by county to estimate the proportion of state customers in each county. We then multiplied the proportion of customers in each county by the estimated total number of customers served in each state from the Energy Information Administration (27) to estimate the number of county customers.”

We’ve also added text to the limitations (page 20, paragraph 3):

“Because POUS did not supply reliable counts of electrical customers by county, we estimated the number of electrical customers using American Community Survey and Energy Information Administration data. We do not know how accurate these estimates are, and, to our knowledge, no better data exist. If these estimates were not accurate, this may have introduced bias (likely non-differential) into our effect estimates.”

(4) If vulnerable individuals (e.g., people of lower SES) are overrepresented among the exposed group, while healthier individuals are not affected, the population-level effect may be overestimated. The authors should clarify how they accounted for this potential bias.

Thank you for bringing up this important point. This is something we considered when conducting this analysis. Our group conducted a study using the same power outage exposure data we use here, and we did find that lower SES counties were likely more exposed to power outage, which means they could be overrepresented among the exposed group. So, it is likely true that the effect we estimate here is influenced by that fact. We’ve included the following sentence in our limitations, which cites our previous study showing that vulnerable communities are likely more exposed (page 20, final paragraph):

“Next, power outages may be more common in high social vulnerability communities (5). High social vulnerability counties may have been over-represented in the exposed group in our study. This means that our effect estimates may not be generalizable to counties with lower social vulnerability, and we may have overestimated the effect of power outage on hospitalization. At the same time, our study population was comprised of Medicare Fee-For-Service beneficiaries, who may be less vulnerable to health effects from power outages due to higher individual-level SES and fewer chronic health conditions compared to Medicare Advantage beneficiaries, which may have led to underestimation of the population-level effect”

(5) Based on Figure 1, most excluded counties are in the Midwest and Western regions, raising concerns about regional representativeness. Please consider focusing on regions with more complete data (e.g., South and Northeast) and assessing whether included and excluded counties are comparable in main characteristics.

We are not certain what the reviewer means by ‘focus on regions with more complete data.’ We think that they may be suggesting that we conduct an additional sensitivity analysis excluding regions with missing data and restricting analyses in those regions of the US with more complete data. However, health-related characteristics and other sociodemographic variables vary so much between regions of the US that if effect estimates from such a sensitivity analysis are different, it would not necessarily be evidence of missing data impacts. There’s no reason *a priori* to think that the effect of outage on health would be the same across regions. Therefore, we have opted not to conduct this additional analysis in this case.

(6) The Poisson regression model was adjusted for temperature, precipitation, and wind speed, but it did not account for air pollution and other extreme weather events, such as wildfires and ice storms, which may influence both power outages and hospitalizations. I suggest adjusting for air pollution and a more comprehensive set of meteorological covariates including extreme weather events in sensitivity analyses.

We appreciate this comment and the opportunity to consider additional important time-varying confounders. After consideration, we do not believe that air pollution confounds the relationship between power outage and healthcare use, other than by operating through extreme weather or wildfire smoke. We have drawn the hypothesized relationships on the DAG below.

**Supplemental Figure 5**: Directed acyclic graph describing hypothesized causal relationships between power outage, hospitalizations among older adults, and potential confounders.

Regarding extreme weather events, we believe that controlling for temperature, precipitation, and wind speed would capture all aspects of these events, such as ice storms, and thus do not pursue additionally adjusting for them.

In response to this comment, we have included a new sensitivity analysis controlling models for smoke days using daily wildfire-generated fine particulate matter estimates from Childs et al. ES&T 2023. While the frequency of co-occurring power outage and wildfire smoke is relatively low, we do agree that when it occurs, wildfire smoke could act as a confounder. We have added new sentences to the methods and results describing this sensitivity analysis (page 9, final paragraph):

“Because of wildfire-related power shutoffs, wildfire smoke could be a confounder. We conducted a sensitivity analysis using estimates of wildfire PM_2.5_ from Childs et al. 2023 to categorize county-days as exposed or unexposed to wildfire smoke. We considered a county-day exposed if the concentration of wildfire smoke was > 0 µg/m³ and adjusted for this binary ‘smoke-day’ variable in our analysis”

To the results, we added (page 13, paragraph 5):

“Our results remained unchanged when we adjusted for wildfire smoke days (**Supplemental Figure 6**).”

≥ 1%

≥ 3%

≥ 5%

**Supplemental Figure 6**: Rate ratios and 95% confidence intervals for the association between county-level 8+ hour power outage exposure and cardiovascular- and respiratory-related hospitalizations in US 2018 Medicare Fee-For-Service beneficiaries for outages affecting ≥1%, ≥3%, and ≥5% of county electrical customers. Estimates are from conditional Poisson regression models adjusted for daily wind speed, temperature, precipitation and wildfire PM_2.5_.

(7) This study did not distinguish between planned and unplanned power outages. Planned outages may have less severe health effects as residents may be better prepared. If possible, the authors should analyze them separately.

This is a very important point – planned and unplanned outages do likely have very different effects on health. Unfortunately, there are no data available that allow us to distinguish between the two at the moment. We have included the following sentence in the limitations section addressing this issue (page 21, paragraph 2):

“Further, we were unable to distinguish between planned and unplanned power outages, which may have different effects on hospitalizations.”

(8) The analyses were conducted at the county level given the exposure assessment method. It would be more appropriate to explore effect modification by county-level characteristics such as urban/rural, socioeconomic status, and geographic region. Since the temperature and season data are readily available, please consider implementing the effect modification by temperature and season in the current study.

Thank you for this comment – we have added these additional analyses to the manuscript.

To the methods, we added the following (page 10, paragraph 2):

“Because power outage may affect health by exposing people to extreme indoor temperatures, we also aimed to test if the effect of power outage on CVD and respiratory hospitalizations changed further on anomalously hot and anomalously cold days. Because we are not aware of existing methods for distributed lags for interactions, we modeled the association between a single lag of power outage exposure and hospitalizations for these effect modification analyses. We selected lags that corresponded to the day with the highest effect estimate for each of our two outcomes. We used the same conditional Poisson model and case-crossover as in the main analysis.

For CVD hospitalizations, we modelled the effect of 8+ hour power outage lagged by two days and same-day anomalously hot temperatures. To test for effect modification on hot days, we included a binary term for anomalously hot days and an interaction term between anomalously hot days and power outage. We conducted a separate analysis to assess for effect modification by cold, including a binary term for same-day anomalously cold days and an interaction term between anomalously cold days and lagged power outage. For respiratory hospitalizations, we took the same approach, but used same-day power outage rather than lagged exposure.

To conduct these analyses, first, we identified anomalously hot and cold days using both a threshold and an absolute cutoff. The temperature on hot days had to fall above both the percentile-based threshold and absolute cutoff and below both for cold days. Using data from 1970-2010, we identified the 15^th^ and 85^th^ percentile temperatures for each county. We then categorized days in our study period (2018) as anomalously hot or cold if they were above the 85^th^ or below the 15^th^ percentile temperatures, *and* hotter than 24^o^C or colder than 0^o^C (absolute cutoffs), respectively.”

To the results, we added the following (page 15, final paragraph):

“When we tested if the effect of power outage on CVD hospitalizations differed on anomalously hot days, we observed a larger power outage-associated increase in CVD hospitalizations on hot days compared to non-hot days (**Supplementary Table 3**). On cold days, there was no additional effect of power outage on CVD hospitalizations. We found no additional effect of power outage on respiratory hospitalization on hot days. The effect of power outage on respiratory hospitalizations was attenuated slightly on anomalously cold days (**Supplementary Table 3**).

We’ve also added the following sentences to the discussion (page 17, final paragraph):

“Further, many power outages are caused by climate-related severe weather like heat waves, winter storms, hurricanes, wildfires, and floods (3, 45, 46), which likely amplifies health risks (20, 47, 48). While we did not directly assess co-exposure to extreme weather in this study, we controlled for temperature, wind, and precipitation as time-varying confounders in our main analysis. We also conducted a secondary analysis where we tested for modification of the effect of power outage on cardiovascular and respiratory hospitalizations by temperature. We analyzed power outages that occurred on anomalously hot or anomalously cold days and found larger effects of power outage on cardiovascular-related hospitalizations on hot days, attenuated effects of power outage on respiratory-related hospitalizations on cold days, and no changes on other days. Enabled by new national datasets of power outage exposure (23, 24), further research could better describe the lagged effects of co-exposure to power outage and extreme temperatures, and could investigate if keeping indoor temperatures stable could prevent some of the adverse health effects of power outages on CVD. Future studies should also examine the joint health effects of outages and severe weather.”

|  | **CVD hot** | **CVD cold** | **Respiratory hot** | **Respiratory cold** |
| --- | --- | --- | --- | --- |
| 8+ hour power outage | RR = 1.03  95% CI: [1.02, 1.04] | RR = 1.04  95% CI: [1.03 1.05] | RR = 1.00  95% CI: [1.00, 1.01] | RR = 1.04  95% CI: [1.02, 1.06] |
| Anomalous temperature day | RR = 1.00  95% CI: [0.99, 1.00] | RR = 1.05  95% CI: [1.04, 1.06] | RR = 1.03  95% CI: [1.01, 1.05] | RR = 1.07  95% CI: [1.06, 1.08] |
| Power outage and anomalous temperature day interaction | RR = 1.06  95% CI: [1.02, 1.10] | RR = 0.96  95% CI: [0.91, 1.01] | RR = 1.02  95% CI: [0.97, 1.07] | RR = 0.91  95% CI: [0.85, 0.97] |

**Supplemental Table 3**: Rate ratios and 95% confidence intervals for the association between county-level 8+ hour power outage exposure and cardiovascular- and respiratory-related hospitalizations in US 2018 Medicare Fee-For-Service beneficiaries on anomalously hot and anomalously cold days. Estimates are from conditional Poisson regression models adjusted for daily wind speed, temperature, and precipitation.

(9) Since the approach to handling missing data is critical to the results, a sensitivity analysis should be conducted to assess the robustness of the findings when removing data with varying proportions of missingness.

We added an additional sensitivity analysis only including counties with less than 20% missing data, in response to the reviewer’s comment. We have included the following additional text in the manuscript, as well as the additional supplemental figure below (sensitivity analyses, page 9, paragraph 2):

“Because of the large proportion of missing data in the POUS dataset, we conducted an additional sensitivity analysis limiting our analysis to counties with 20% of data missing or less”

And to the results, we added (results, sensitivity analyses, page 12): “Restricting to counties with 80% or higher coverage (compared to 50% or higher in our main analyses) did not change our results (**Supplemental Figure 4**).”

**Supplemental Figure 4**: Rate ratios and 95% confidence intervals for the association between county-level 8+ hour power outage exposure and cardiovascular- and respiratory-related hospitalizations in US 2018 Medicare Fee-For-Service beneficiaries for outages affecting ≥1%, ≥3%, and ≥5% of county electrical customers, in counties with 20% of data missing or less. Estimates are from conditional Poisson regression models adjusted for daily wind speed, temperature, and precipitation.

≥ 1%

≥ 3%

≥ 5%

Minor comments:
(1) Please consider including the results for qAIC in supplementary materials for completeness.

Yes, thank you - we’ve added the qAICs to the supplement.

(2) In the main analysis results for CVD hospitalizations, the authors report increases in CVD-related hospitalizations 1-3 and 6 days after power outage exposure. However, Figure 2 suggests that significant increases are only observed on lag days 1 and 2. Additionally, Figure 2 indicates that power outages with a size ≥1% appear to have protective effects on CVD-related hospitalizations on lag days 4 and 5 (a similar pattern is observed in Supplemental Figure 1). Could the authors provide an explanation for these findings?

Thank you for bringing this up. There is some evidence in the literature of week-later delayed effects of heat on CVD hospitalizations, which is why we interpreted lag 6 as positive, but we were already on the fence about how to interpret that last lag, even before comments from both this reviewer and Reviewer 1 about lag 6. We have removed that interpretation from our manuscript, and it now reads (page 12, CVD hospitalizations, Main analysis):

“In our main analysis, we used a case-crossover design with a conditional Poisson model to test the association between 8+ hour power outage and emergency CVD hospitalization rates up to 1 week after power outage exposure. We found increases in CVD-related hospitalizations 1-3 days after power outage exposure (**Figure 2**)”

The observed protective effect on hospitalizations 4–5 days after a power outage may be a harvesting effect, or an artifact produced by using a natural spline-based model. For larger outages, there seems to be no protective effect on hospitalizations 4-5 days after exposure, making it unclear to us whether any true protective effect exists. We’ve added the following text explaining our interpretation of these findings to the discussion (page 17, paragraph 1):

“We observed a protective effect on hospitalizations 4–5 days after a power outage, which may be a harvesting effect or an artifact resulting from the use of a natural spline-based model. For larger outages, there was no protective effect on hospitalizations 4-5 days after exposure, making it unclear whether any true protective effect exists.”

(3) The discussion could include an explanation for the differing lag effects observed for CVD and respiratory-related hospitalizations.

Yes – we chose to analyze these two outcomes separately because we hypothesized that the effects of power outage on CVD and respiratory hospitalizations may be different. We have included the following text in the discussion (page 17, paragraph 2):

“We hypothesized that power outages may lead to CVD and respiratory hospitalizations in older adults due to increased heat exposure, cold exposure, stress, and loss of electricity to life-sustaining medical devices and mobility aids. Power outages may also cause changes in indoor air quality when dehumidifiers, air purifiers, and ventilation systems lose power. The lagged effects of CVD and respiratory hospitalizations due to power outage likely differ, since the lagged effects of high or low temperature and air pollution appear to differ for CVD and respiratory disease (37, 43, 44).”

Thank you for your comments Reviewer 3!

Editor comments:

1. Agree with R2 about improving the description of the total number of outages (7 outages, SD=29, of 8+ hours). The huge right skew renders mean/SD uninterpretable, so median/IQR is needed.

We have changed this to report the IQR and 5^th^ and 95^th^ quantiles: (page 11, Results, paragraph 2).

“We included 2,161 US counties, covering 71.1% of older adult Medicare Fee-For-Service beneficiaries aged 65+ (N = 23,645,101). These counties experienced a median of 2 (IQR = 4, 5^th^ percentile = 0, 95^th^ percentile = 15) 8+ hour power outages affecting ≥1% of customers in 2018 (**Figure 1**).”

2. Also, even if removing the mean/SD, as R2 points out I just think the phrasing of this statistic is not easy for readers: "counties experienced an average of 7 (standard deviation, SD=29) 8+ hour power outages affecting ≥1% of customers in 2018." I have lived in rural U.S., and I had maybe 1 of these outages per year. So it does not resonate with me. The issue is definitional -- the reader's mind automatically interprets this to mean that each individual in the U.S> experiences 8 outages per year. But the 1% bit of the statistic is doing a lot of work here -- it means that only a tiny number of households, different each time, are affected. The more useful statistic is the median + IQR of power outages experienced by a given person/household per year.

Thanks for highlighting this point again. What you said makes a lot of sense. We also wanted to calculate the statistic that you mention, but we cannot with the data we have available. We have updated the text to help the reader interpret our exposure metric (page 11, Results, paragraph 2):

“We included 2,161 US counties, covering 71.1% of older adult Medicare Fee-For-Service beneficiaries aged 65+ (N = 23,645,101). These counties experienced a median of two (IQR = 4, 5^th^ percentile = 0, 95^th^ percentile = 15) 8+ hour power outages affecting ≥1% of customers in 2018 (**Figure 1**). Because counties were considered exposed to an 8+ hour power outage if ≥1% of customers were without power for 8+ hours, it was not the case that individuals experienced a median of two 8+ hour outages; rather, a small portion of customers in these counties were likely affected by 8+ hour outages.”

3. Would a DAG be helpful in illuminating the assumptions in this causal inference paper? In particular, being transparent about which confounders are and are not controlled for?

Yes – we can make a DAG! Here it is:

**Supplemental Figure 5**: Directed acyclic graph describing hypothesized causal relationships between power outage, hospitalizations among older adults, and potential confounders.

We controlled for wind speed, temperature, and precipitation, which we think should capture severe weather and its impact on hospitalizations. Those are the variables in the top right. Our case-crossover study design automatically controls for the county-level characteristics in the top-left corner that could also confound the relationship of interest.

We have added this to DAG the supplemental content.

4. Could the authors describe their results in absolute terms? The Poisson output of IRR is useful as a relative measure. But ultimately, it is hard to get a sense for the absolute impact as all of the IRRs are tiny even if statistically significant.

Yes – we’ve added the following to the methods and the results:

Methods (page 9, paragraph 2):

“Finally, to express our main analysis results in absolute terms, we calculated the population attributable fraction of cardiovascular and respiratory hospitalizations due to power outage and used it to estimate the excess cardiovascular and respiratory hospitalizations associated with power outages in the Medicare Fee-For-Service population in 2018.”

Results (page 16, paragraph 2):

“With an average of seven 8+ hour power outages per year, a 1.04 RR (95% CI: 1.02, 1.06) increase in CVD-related hospitalizations in the seven-day period following an outage would result in approximately 2408 (95% CI: 1105, 3735) CVD-related hospitalizations due to power outage per year in the Fee-for-Service Medicare population, in the absence of other sources of bias. Similarly, across all lags, a 1.05 RR (1.02, 1.08) increase in respiratory-related hospitalizations with power outage would result in approximately 1838 (95% CI: 819, 2884) power outage-attributable respiratory hospitalizations per year in this population.”
