# Supplementary material for: The association between power outages and cardiovascular and respiratory hospitalizations among US Medicare beneficiaries in 2018: A case-crossover study
Source: PLoS Med. 2026 Mar 12;23(3):e1004923. doi: 10.1371/journal.pmed.1004923 (PMC12994585; doi:10.1371/journal.pmed.1004923)
Supplement: Attachment — Submitted filename: editorial_requests_oct_26.docx [file pmed.1004923.s013.docx]

PLOS Medicine editorial requests

Thank you for your detailed read of our manuscript!

* At this stage, we ask that you include a short, non-technical Author Summary of your research to make findings accessible to a wide audience that includes both scientists and non-scientists. The Author Summary should immediately follow the Abstract in your revised manuscript. This text is subject to editorial change and should be distinct from the scientific abstract. Ideally each sub-heading should contain 2-3 single sentence, concise bullet points containing the most salient points from your study. In the final bullet point of ‘What Do These Findings Mean?’ Please include the main limitations of the study in non-technical language.

Here is our author summary:

**Why Was This Study Done? Authors should reflect on what was known about the topic before the research was published and why the research was needed.**

- Power outages threaten health when air conditioners, heaters, communication devices like phones and laptops, and medical devices like at-home ventilators and oxygen tanks lose power, exposing people to extreme temperatures, causing medical and mobility device failures, and isolating individuals from help
- Prior studies have shown power outages might increase risk of emergency department visits and hospitalizations because of this heat, cold, medical device failure, and isolation
- Power outages are becoming more common with climate change, electricity use by artificial intelligence, and electrification, so understanding the health risks of power outages is important

**What Did the Researchers Do and Find? Authors should briefly describe the study design that was used and the study’s major findings. Do include the headline numbers from the study, such as the sample size and key findings.**

- We estimated risk of hospitalization for older adults (age 65+) on days with 8+ hour power outages compared to days without power outage
- We found that power outages lasting 8+ hours were associated with an increased risk of hospitalization for cardiovascular and respiratory disease
- Assuming power outages are causing these hospitalizations, we estimated that there were 4500 excess hospitalizations in 2018 among US adults 65+ due to power outages

**What Do These Findings Mean? Authors should reflect on the new knowledge generated by the research and the implications for practice, research, policy, or public health. Authors should also consider how the interpretation of the study’s findings may be affected by the study limitations.**

- Power outages may put older adults at risk of hospitalization for cardiovascular and respiratory issues because of heat and cold, medical device failure, and social isolation
- Investing in electricity reliability could improve community health, as could other interventions like backup batteries for medical devices and cellphones and laptops, or generators to keep air conditioners and heaters on, especially as electrification continues

* Please confirm that your title complies with to PLOS Medicine's style. Your title must be nondeclarative and not a question. It should begin with main concept if possible. "Effect of" should be used only if causality can be inferred, i.e., for an RCT. Please place the study design ("A randomized controlled trial," "A retrospective study," "A modelling study," etc.) in the subtitle (ie, after a colon).

We updated our title to conform with the guidelines:

“Power outages and cardiovascular and respiratory hospitalizations among US Medicare beneficiaries in 2018: a case-crossover study”

* Please revise your abstract to comply with our requirements, including format (three sections: Background, Methods and Findings, and Conclusions) and providing all the information relevant to this study type

We have updated the abstract sections.

* Please ensure that the Introduction ends with a clear description of the study question or hypothesis.

We’ve confirmed that we did this. The last paragraph of our intro reads:

We previously assembled the first nationwide dataset of hourly county-level power outage exposure from 2018-2020 based on data from PowerOutage.us (5, 24). Here, we leveraged these data together with 2018 older adult Medicare Fee-For-Service hospitalization claims to estimate the relationship between daily county-level power outage exposure and cardiovascular and respiratory hospitalization rates in the United States in a case-crossover study. We evaluated the impacts of moderate and large-scale power outages and conducted secondary analyses examining effect measure modification by age, sex, Medicaid eligibility, and electricity-dependent durable medical equipment (DME) use.

* Please ensure that all abbreviations are defined at first use throughout the text.

We’ve confirmed that we have defined all abbreviations.

* Please confirm that all numbers presented in the abstract are present and identical to numbers presented in the main manuscript text.

We’ve confirmed that all our numbers are correct.

* Please review your text for claims of novelty or primacy (e.g. 'for the first time') and remove this language. In addition, please check that any use of statistical terms (such as trend or significant) are supported by the data, and if not please remove them.

We’ve removed all claims of novelty.

* Please remove the 'conclusions' subheading from the discussion. Please also remove any other subheadings from the discussion.

We’ve removed all subheadings from the discussion.

* In the last sentence of the Abstract Methods and Findings section, please describe the main limitation(s) of the study's methodology.

We have added a sentence about the limitations of our exposure data.

* In the abstract, please include the important dependent variables that are adjusted for in the analyses.

We have confirmed we included confounders in our abstract.

* The Data Availability Statement (DAS) requires revision. For each data source used in your study: 

a) If the data are freely or publicly available, note this and state the location of the data: within the paper, in Supporting Information files, or in a public repository (include the DOI or accession number).

b) If the data are owned by a third party but freely available upon request, please note this and state the owner of the data set and contact information for data requests (web or email address). Note that a study author cannot be the contact person for the data.

c) If the data are not freely available, please describe briefly the ethical, legal, or contractual restriction that prevents you from sharing it. Please also include an appropriate contact (web or email address) for inquiries (again, this cannot be a study author).
* Please indicate how you obtained Medicare information if not available to other researchers.

We have added more detail to our data availability statement:

Medicare beneficiary data are available from https://data.medicare.gov/ for researchers who meet the criteria for access to confidential data.

* Please explain whether you purchased the power outage data.

We added a sentence clarifying that we did purchase those data:

“To assess power outage exposure for 2018, we used nationwide county-level data purchased from PowerOutage.us (POUS) (24).”

* Please include the statement on code availability and url in the data availability statement.

We’ve added that to the data availability statement.

* Please provide the name(s) of the institutional review board(s) that provided ethical approval as well as the application identifier.

We’ve updated the IRB statement to the following:

“This study was approved by the Institutional Review Boards at WCG (single IRB for multi-site project 20210419), the Harvard T.H. Chan School of Public Health, and the Columbia University Mailman School of Public Health.”

* Please confirm that the appropriate usage rights apply to the use of this map. Please see our guidelines for map images: <https://nam02.safelinks.protection.outlook.com/?url=https%3A%2F%2Fjournals.plos.org%2Fplosmedicine%2Fs%2Ffigures%23loc-maps&data=05%7C02%7Chm2913%40cumc.columbia.edu%7C688fc3c89bd3471cd69e08de0dc9fc89%7Cb0002a9b0017404d97dc3d3bab09be81%7C0%7C0%7C638963358349956842%7CUnknown%7CTWFpbGZsb3d8eyJFbXB0eU1hcGkiOnRydWUsIlYiOiIwLjAuMDAwMCIsIlAiOiJXaW4zMiIsIkFOIjoiTWFpbCIsIldUIjoyfQ%3D%3D%7C0%7C%7C%7C&sdata=9aj%2BV%2F4aQlZ7u%2BJRpGEaq8%2FoC7QZZHGTjTQb8yleo8c%3D&reserved=0> . Also note that figures cannot be reproduced from other sources that are not CC-BY.

This map was created using publicly available census data in R, so we do have appropriate usage rights.

* Please provide the unadjusted comparisons as well as the adjusted comparisons in all relevant Tables

We do not have any tables that have adjusted comparisons.

* Please specify the variables controlled for in all relevant Tables.

We controlled for variables in analyses presented in supplemental tables 2 and 3. We specified the variables controlled for in these table captions:

**“Supplemental Table 2**: Rate ratios and 95% confidence intervals for the association between county-level 8+ hour power outage exposure and CVD and respiratory hospitalizations in US 2018 Fee-For-Service Medicare beneficiaries for outages affecting ≥1%, ≥3%, and ≥5% of county electrical customers. Estimates are from conditional Poisson regression models adjusted for daily wind speed, temperature, and precipitation.

**Supplemental Table 3**: Rate ratios and 95% confidence intervals for the association between county-level 8+ hour power outage exposure and cardiovascular- and respiratory-related hospitalizations in US 2018 Medicare Fee-For-Service beneficiaries on anomalously hot and anomalously cold days. .Estimates are from conditional Poisson regression models adjusted for daily wind speed, temperature, and precipitation.”

OK. We’ll quote where we did this.

*Please revise your references as per PLOS Medicine style.

We have updated our references to PLOS Medicine style.

* The funding statement should include: specific grant numbers, initials of authors who received each award, URLs to sponsors’ websites. Also, please state whether any sponsors or funders (other than the named authors) played any role in study design, data collection and analysis, the decision to publish, or preparation of the manuscript. If they had no role in the research, include this sentence: “The funders had no role in study design, data collection and analysis, decision to publish, or preparation of the manuscript.”

We’ve updated our funding statement to include websites.

* Your study is observational and therefore causality cannot be inferred. Please remove language that implies causality and refer to associations instead. Please revise the Abstract accordingly.

We have confirmed we refer to associations in the abstract and text.

For Observational studies:
* Please ensure that the study is reported according to the STROBE guideline, and include the completed STROBE checklist as Supporting Information. Please add the following statement, or similar, to the Methods: "This study is reported as per the Strengthening the Reporting of Observational Studies in Epidemiology (STROBE) guideline (S1 Checklist)."

The STROBE guideline can be found here: <https://nam02.safelinks.protection.outlook.com/?url=http%3A%2F%2Fwww.equator-network.org%2Freporting-guidelines%2Fstrobe%2F&data=05%7C02%7Chm2913%40cumc.columbia.edu%7C688fc3c89bd3471cd69e08de0dc9fc89%7Cb0002a9b0017404d97dc3d3bab09be81%7C0%7C0%7C638963358349982468%7CUnknown%7CTWFpbGZsb3d8eyJFbXB0eU1hcGkiOnRydWUsIlYiOiIwLjAuMDAwMCIsIlAiOiJXaW4zMiIsIkFOIjoiTWFpbCIsIldUIjoyfQ%3D%3D%7C0%7C%7C%7C&sdata=aR35wnft1tKWyhHVpdsKfltN%2FUh%2BWT6w4NPkk5po4%2FU%3D&reserved=0>

When completing the checklist, please use section and paragraph numbers, rather than page numbers.

We completed the RECORD guideline since it includes STROBE.

* Did your study have a prospective protocol or analysis plan? Please state this (either way) early in the Methods section.

a) If a prospective analysis plan (from your funding proposal, IRB or other ethics committee submission, study protocol, or other planning document written before analyzing the data) was used in designing the study, please include the relevant prospectively written document with your revised manuscript as a Supporting Information file to be published alongside your study, and cite it in the Methods section. A legend for this file should be included at the end of your manuscript. 

b) If no such document exists, please make sure that the Methods section transparently describes when analyses were planned, and when/why any data-driven changes to analyses took place.

We did not have a publishable analysis plan, but have confirmed that our Methods section transparently describes which analyses were planned. We did not conduct any unplanned data-driven analyses.

c) In either case, changes in the analysis-- including those made in response to peer review comments-- should be identified as such in the Methods section of the paper, with rationale.

We made no changes in the analysis.

* For all observational studies, in the manuscript text, please indicate: (1) the specific hypotheses you intended to test, (2) the analytical methods by which you planned to test them, (3) the analyses you actually performed, and (4) when reported analyses differ from those that were planned, transparent explanations for differences that affect the reliability of the study's results. If a reported analysis was performed based on an interesting but unanticipated pattern in the data, please be clear that the analysis was data-driven.
Please refer to the following guidance for Population Health/Registry studies:
Please ensure that the study is reported according to the RECORD guideline (available from <https://nam02.safelinks.protection.outlook.com/?url=https%3A%2F%2Fwww.record-statement.org%2F&data=05%7C02%7Chm2913%40cumc.columbia.edu%7C688fc3c89bd3471cd69e08de0dc9fc89%7Cb0002a9b0017404d97dc3d3bab09be81%7C0%7C0%7C638963358349999603%7CUnknown%7CTWFpbGZsb3d8eyJFbXB0eU1hcGkiOnRydWUsIlYiOiIwLjAuMDAwMCIsIlAiOiJXaW4zMiIsIkFOIjoiTWFpbCIsIldUIjoyfQ%3D%3D%7C0%7C%7C%7C&sdata=uWhFjS4qLf2r%2FHqS%2BwXQdrDJRVxWogcszd1pWEFGKiA%3D&reserved=0>) and include the completed checklist as Supporting Information. Please add the following statement, or similar, to the Methods: "This study is reported as per the Reporting of Studies Conducted using Observational Routinely-Collected Data (RECORD) guideline (S1 Checklist)." When completing the checklist, please use section and paragraph numbers, rather than page numbers.

We have included a RECORD checklist as a supplementary document.

Please refer to the following guidance for Population-level Health Estimates:
* Please report your data according to GATHER and enclose a completed GATHER checklist as a supplementary document. See <https://nam02.safelinks.protection.outlook.com/?url=http%3A%2F%2Fgather-statement.org%2F&data=05%7C02%7Chm2913%40cumc.columbia.edu%7C688fc3c89bd3471cd69e08de0dc9fc89%7Cb0002a9b0017404d97dc3d3bab09be81%7C0%7C0%7C638963358350018858%7CUnknown%7CTWFpbGZsb3d8eyJFbXB0eU1hcGkiOnRydWUsIlYiOiIwLjAuMDAwMCIsIlAiOiJXaW4zMiIsIkFOIjoiTWFpbCIsIldUIjoyfQ%3D%3D%7C0%7C%7C%7C&sdata=gZ5ViAKO2Kz6cmSSLB0yDxau6tFwghOPMz9toRVOLPs%3D&reserved=0> In the checklist please include sufficient text excerpted from the manuscript to explain how you accomplished all applicable items.

We did not address the comments below because did not conduct a modelling study.

For modeling studies, we request that the following guidance be followed (derived from Geoffrey P Garnett, Simon Cousens, Timothy B Hallett, Richard Steketee, Neff Walker. Mathematical models in the evaluation of health programmes. (2011) Lancet DOI:10.1016/S0140-6736(10)61505-X.):
* Please provide a diagram that shows the model structure, including how the disease natural history is represented, the process and determinants of disease acquisition, and how the putative intervention could affect the system.
* Please provide a complete list of model parameters, including clear and precise descriptions of the meaning of each parameter, together with the values or ranges for each, with justification or the primary source cited, and important caveats about the use of these values noted.
* Please provide a clear statement about how the model was fitted to the data including where relevant goodness-of-fit measure, the numerical algorithm used, which parameter varied, constraints imposed on parameter values, and starting conditions.
* For uncertainty analyses, please state the sources of uncertainties quantified and not quantified this can include parameter, data, and model structure.
* Please provide sensitivity analyses to identify which parameter values are most important in the model. Uncertainty estimates seek to derive a range of credible results on the basis of an exploration of the range of reasonable parameter values. The choice of method should be presented and justified.
* Please discuss the scientific rationale for this choice of model structure and identify points where this choice could influence conclusions drawn. Please also describe the strength of the scientific basis underlying the key model assumptions.

We did not conduct a modeling study.
