# Supplementary material for: The association between power outages and cardiovascular and respiratory hospitalizations among US Medicare beneficiaries in 2018: A case-crossover study
Source: PLoS Med. 2026 Mar 12;23(3):e1004923. doi: 10.1371/journal.pmed.1004923 (PMC12994585; doi:10.1371/journal.pmed.1004923)
Supplement: Attachment — Submitted filename: editorial_comments_response_nov_10.docx [file pmed.1004923.s014.docx]

* Author Summary: please add a final sentence to your summary stating briefly the study limitations.

We have added the following sentence:

“The data used in this study only described power outages by county, instead of for individual people. In the future, knowing which individuals experienced a power outage can help us understand the exact risks of power outage for health.”

* Title: we would suggest starting the title as follows ‘The association between power outages…

We have changed it to “The association between power outages and cardiovascular and respiratory hospitalizations among US Medicare beneficiaries in 2018: a case-crossover study”

* Abstract: please revise the heading Conclusion to Conclusions.

We have fixed this.

*Please remove significance statement from the manuscript.

We have removed this.

* Please make the hypotheses tested clearer at the end of the introduction section, i.e. we tested if power outages were associated with emergency CVD and respiratory disease-related hospitalizations among older adults in the United States.

We changed the final paragraph to the following:

“We previously assembled a nationwide dataset of hourly county-level power outage exposure from 2018-2020 based on data from PowerOutage.us [5,24]. Here, we used these data together with 2018 older adult Medicare Fee-For-Service hospitalization claims to test if daily county-level power outage exposure was associated with emergency cardiovascular and respiratory hospitalization rates among older adults in the United States, using a case-crossover design. We evaluated the impacts of moderate and large-scale power outages and conducted secondary analyses examining effect measure modification by age, sex, Medicaid eligibility, and electricity-dependent durable medical equipment (DME) use. We hypothesized power outages would cause cardiorespiratory hospitalizations among older adults, and risks would be higher among oldest and Medicaid-eligible adults, and in counties with higher DME use.”

* Abbreviations – Please define on first use WCG, IQR, COPD, ZCTA, SES, ICD, PM, CI.

We have defined all these abbreviations.

* Please check that any use of statistical terms (such as trend or significant) are supported by the data, and if not please remove them.

We have checked that we didn’t use these words.

* Please revise your Data Availability Statement (DAS) and ensure it includes for each data set used in your study:
a) If the data are owned by a third party but freely available upon request, please note this and state the owner of the data set and contact information for data requests (web or email address). Note that a study author cannot be the contact person for the data.

We have added this in the submission system.

b) If the data are not freely available, please describe briefly the ethical, legal, or contractual restriction that prevents you from sharing it. Please also include an appropriate contact (web or email address) for inquiries (again, this cannot be a study author).

We have added this in the submission system.

* Please also include the statement on code availability and url in the data availability statement.

We have added this in the submission system.

*Please add IRB approval numbers to the ethics statement and indicate the form of consent obtained (written/verbal) or the reason that consent was not obtained (e.g. the data were analyzed anonymously).

We have added this in the submission system.

*Please ensure that the study is reported according to the RECORD guideline (available from <https://nam02.safelinks.protection.outlook.com/?url=https%3A%2F%2Fwww.record-statement.org%2F&data=05%7C02%7Chm2913%40cumc.columbia.edu%7C80b68e1de356413cb39c08de20a3bb46%7Cb0002a9b0017404d97dc3d3bab09be81%7C0%7C0%7C638984084775229261%7CUnknown%7CTWFpbGZsb3d8eyJFbXB0eU1hcGkiOnRydWUsIlYiOiIwLjAuMDAwMCIsIlAiOiJXaW4zMiIsIkFOIjoiTWFpbCIsIldUIjoyfQ%3D%3D%7C0%7C%7C%7C&sdata=kEm5Sc9kPl1vqEvRYETSNnkZA8d6SxGvP%2BIdhLc9%2Fjo%3D&reserved=0>) and include the completed checklist as Supporting Information. Please add the following statement, or similar, to the Methods: "This study is reported as per the Reporting of Studies Conducted using Observational Routinely-Collected Data (RECORD) guideline (S1 Checklist)." When completing the checklist, please use section and paragraph numbers, rather than page numbers. (edited)

We have added this statement to the end of the methods section, and we’ve included the checklist.
